# Latent profiling of five-dimensional psychological resilience across generations: a deep clustering and behavioural divergence analysis in pre-conflict Iran

**DOI:** 10.3389/fpsyt.2025.1669687

**Published:** 2026-01-15

**Authors:** Taghi Shakouri Youvalari, Inci Zaim Gökbay

**Affiliations:** 1 Institute of Graduate Studies in Sciences, Informatics, Istanbul University, Istanbul, Türkiye; 2 Department of Artificial Intelligence and Data Engineering, Faculty of Computer and Information Technologies, Istanbul University, Istanbul, Türkiye

**Keywords:** behavioural drift, deep clustering, generational analysis, internal tension, Iranian society, machine learning in mental health, psychological resilience, resilience archetypes

## Abstract

Psychological resilience is increasingly conceptualized as a multidimensional construct encompassing identity, emotional, cognitive, behavioural, and social domains. Using data from 620 Iranian adults (aged 18–64 years; 52% female), collected through an online self-report survey, this study applied unsupervised machine-learning techniques combining a deep autoencoder for dimensionality reduction with a Gaussian Mixture Model (GMM) for latent clustering-to examine psychological resilience profiles in pre-conflict Iran. Thirty-seven standardized psychological subscales were aggregated into five theoretically grounded dimensions: Self-Identity and Meaning, Emotional Regulation, Cognitive Flexibility, Coping and Growth, and Social Support and Connectedness. Unsupervised analysis identified four latent archetypes-Fragile Striver, Reactive Idealist, Hidden Reactor, and Stable Withdrawer-that reflected nonlinear configurations of resilience capacities across generational and gender groups. However, because the research employed a cross-sectional and self-report design, findings illustrate associative rather than causal relationships, and representativeness is limited to online participants. These contextual and demographic influences suggest that resilience is embedded within Iran’s evolving social environment. Despite these limitations, the study demonstrates the potential of AI-based latent profiling to clarify the multidimensional nature of resilience within culturally demanding contexts.

## 1 Introduction

Psychological resilience-the ability to adapt to stress while maintaining mental and emotional stability-is increasingly understood as a dynamic and context-sensitive process. In societies facing long-term adversity, such as Iran, understanding how resilience varies across individuals and generations holds both scientific and practical value. Much of the existing research focuses on stable personality traits or clinical populations, which can obscure the more nuanced, shifting patterns of coping seen in the general population. This study addresses that gap by exploring hidden psychological resilience profiles in a non-clinical Iranian sample. Drawing on a multidimensional framework and unsupervised machine learning, we aim to identify latent patterns that show how individuals adapt to ongoing systemic stress. The next section outlines the cultural and generational background that frames the study.

### 1.1 Contextual background

Psychological resilience is a key factor in how individuals adapt to stress and uncertainty. In societies that face repeated social, political, or economic pressures, understanding resilience becomes especially important. Iran provides a relevant context for examining resilience under chronic systemic stress. Over the past four decades, Iranians have experienced the effects of war, international sanctions, political restrictions, and economic instability (1, 2). These long-term pressures are associated with distinctive coping processes such as strengthened emotional regulation, flexible cognitive appraisal, and reliance on collective social support systems (3, 4). Such contextual conditions highlight the relevance of examining resilience not as a fixed trait but as an adaptive system of interrelated psychological domains. Beyond structural hardship, Iranian cultural values-family cohesion, spirituality, and communal responsibility-serve as protective factors that foster meaning-making and collective endurance (5). Resilience in this sociocultural context often emerges through religious reflection, inter-generational solidarity, and social modesty norms that encourage emotional restraint and mutual support. These features give Iranian resilience a culturally grounded character distinct from Western individualistic conceptions.

Empirical studies in Iranian samples further indicate that coping styles and self-efficacy are shaped by contextual and generational experiences (1, 3). Such evidence supports viewing resilience as a multidimensional construct encompassing self-identity, emotional regulation, cognitive flexibility, coping and growth, and social connectedness. Younger Iranians, typically aged 18–35 and raised in an era of digital connectivity and global exposure, face internal limits related to expression, mobility, and opportunity (6). In contrast, older generations-often 50 years and aboveretain memories of war, post-revolution transformation, and restricted civic participation (5). This generational divide may contribute to differences in emotional regulation, uncertainty management, and the formation of identity within collective family structures. Together, these demographic contrasts illustrate how resilience strategies are negotiated within shared cultural expectations yet differentiated by life stage and social experience.

Despite the recognized importance of these dynamics, few studies have examined resilience across an entire population living under prolonged socio-political strain, and even fewer have integrated sociocultural and generational perspectives in doing so (4, 7). This gap limits our understanding of how Iranian people sustain psychological functioning under continuous pressure. These contextual foundations are revisited in the Discussion when interpreting generational resilience archetypes and cultural variations in adaptive profiles. Taken together, the conditions outlined here underscore the need for a multidimensional framework capable of capturing how resilience functions across interacting psychological domains, providing a basis for the conceptual model introduced in the next section.

### 1.2 Conceptual framing of resilience

Building on the sociocultural and generational context outlined above, resilience is the capacity to adapt effectively to adversity or major life changes. It represents a dynamic system of emotional, cognitive, and social processes that maintain psychological functioning under stress (8, 9). Contemporary work emphasizes interacting mechanisms-selfregulation, meaning-making, problem-solving, and social connectedness-that jointly sustain adaptation (10, 11). Resilience-related identity processes are closely linked to continuity with the future self, which influences long-term decision-making (12), and recent studies show that latent profile analysis can effectively capture heterogeneous resilience patterns across populations (13).

Resilience theory has increasingly adopted multidimensional models, viewing adaptation as a pattern of interacting domains rather than a single trait. Kalisch et al. (14) and Fletcher and Sarkar (15) showed that emotional regulation, cognitive flexibility, and social support operate together to protect mental health. This evidence supports frameworks that go beyond single-scale assessments (9, 11).

The current study synthesizes these findings into a five-dimensional (5D) model integrating validated subscales from established measures (e.g., CD-RISC, Brief COPE). The domains include: (1) Self-Identity and Meaning, (2) Emotional Regulation, (3) Cognitive Flexibility, (4) Coping and Growth Strategies, and (5) Social Support and Connectedness. These domains capture complementary aspects of adaptive functioning validated in prior research (10, 14).

In the Iranian context, these dimensions appear through culturally embedded practices: meaning is tied to family and spirituality, coping draws on shared problem-solving, and social support emerges through extended kinship networks (3, 5). Generational differences also shape these dimensions-young adults emphasize cognitive flexibility, while older adults rely on spiritual and collective endurance (6). Cross-cultural evidence confirms these multidimensional factors across societies (16, 17). This 5D framework thus provides the theoretical basis for the latent-profiling analysis described in the Methods and revisited in the Discussion.

### 1.3 Research problem and gap

Although psychological resilience has been widely studied, most existing work focuses on clinical or trauma-exposed populations and relies on narrow, trait-based assessments. Such approaches capture only a fragment of adaptive functioning and overlook the dynamic, multidimensional nature of resilience expressed in everyday life. In contexts like Iran-where long-term social, economic, and political pressures shape how people cope-understanding these adaptive processes provides critical insight into collective psychological health.

In Iran, many individuals face chronic stress arising from economic hardship, restricted social freedoms, and intergenerational tension. Yet prior studies have concentrated on specific symptoms or isolated coping strategies (1, 4). Few investigations have examined the integrated, multidimensional structure of resilience across non-clinical populations, and even fewer have explored how these adaptive configurations differ between younger and older generations with distinct life experiences and social exposures (6, 7). Comparing these generations reveals how shifting cultural norms and collective memories produce contrasting adaptive mechanisms-an important dimension for understanding social change.

Empirical studies employing data-driven methods remain scarce. Most research relies on predefined categories or mean-score comparisons, potentially masking nuanced patterns within populations. Recent advances in psychology and computational science show that unsupervised learning can detect latent psychological patterns overlooked by classical statistics (18, 19). However, these techniques have rarely been applied in non-Western cultural settings or integrated with multidimensional theoretical frameworks. Iran therefore offers a unique testing ground, where cumulative stress and strong collectivist values produce distinctive resilience profiles.

This study addresses these gaps by integrating a five-dimensional resilience framework with autoencoder-based dimensionality reduction and Gaussian Mixture clustering to identify latent psychological profiles within a non-clinical community sample (N = 620). The goal is not to infer causality but to explore patterns of association between resilience dimensions and generational differences. Demographic variables such as gender and education are also considered to contextualize these profiles. By combining theoretical and computational approaches, this research provides one of the first latent-profiling analyses of resilience in a non-Western setting and demonstrates the potential of machine learning to enrich psychological -Modelling. The following section describes the sample, instruments, and analytical procedures used to examine these latent structures.

### 1.4 Study aim and innovation

This study aims to identify and interpret latent psychological resilience archetypes in a nonclinical Iranian population using a five-dimensional (5D) framework grounded in established theory. The five dimensions-Self-Identity and Meaning, Emotional Regulation, Cognitive Flexibility, Coping and Growth Strategies, and Social Support and Connectedness-represent complementary components of adaptive functioning. Each dimension was operationalized through validated psychometric subscales that together capture the multidimensional structure of resilience. The goal is to explore how these dimensions combine across individuals and how their configurations vary by age and sociocultural experience. This approach allows resilience to be examined within Iran’s collectivist context, where shared social values and intergenerational experiences strongly influence adaptive behaviour.

To identify hidden psychological patterns, a deep autoencoder was used for nonlinear dimensionality reduction, producing latent feature representations that served as inputs for Gaussian Mixture Model (GMM) clustering.

GMM was selected as the final method because it accommodates continuous latent distributions and probabilistic membership, providing greater interpretive flexibility than partition-based algorithms such as K-Means. This integration of computational -Modelling and cultural psychology offers insight into resilience patterns unique to Iran’s evolving social environment.

Two exploratory indices- behavioural drift and internal tension-were developed to enhance interpretation. Behavioural drift refers to the variability of an individual’s responses across resilience dimensions, whereas internal tension captures discrepancies between personal resources and emotional stability. These measures help reveal whether an archetype reflects cohesive or fragmented adaptation All analyses were performed in Python 3.11 using TensorFlow 2.14 and scikit-learn 1.3 with fixed random seeds to ensure reproducibility By combining theoretical modelling, data science, and cultural interpretation, this study provides an empirically grounded integration of psychological theory and unsupervised learning rather than claiming a wholly new paradigm. The resulting resilience archetypes reflect generational differences embedded within shared cultural narratives, informing how age-related experiences shape adaptive capacity. These innovations guide the analyses and profile interpretations presented in the subsequent Methods and Results sections.

### 1.5 Research questions

Grounded in the five-dimensional (5D) resilience framework and supported by unsupervised learning techniques, this study explores how multidimensional psychological resources combine to shape adaptive functioning in Iranian adults.

The research questions derive directly from the theoretical dimensions and their psychometric indicators, ensuring alignment between conceptual and empirical design. They also reflect Iran’s sociocultural environment, where generational differences and collective values influence patterns of resilience.

RQ 1. What latent psychological resilience archetypes can be identified through autoencoderbased feature extraction combined with Gaussian Mixture Model (GMM) clustering, and how are these archetypes distributed across the five resilience dimensions? Which patterns of behavioural drift (within-profile variability) and internal tension (inconsistency between personal resources and emotional stability) characterize each archetype?

RQ 2. In what ways do these archetypes vary across age cohorts representing different sociocultural generations in Iran? Are specific archetypes more prevalent in one group, and how do they differ in the relative balance of the five dimensions?

RQ 3. Which psychometric subscales or dimension-specific features most effectively predict membership in each archetype? Can machine-learning classifiers accurately differentiate adaptive from less adaptive profiles, and which features contribute most to model performance? RQ 4. How can the identified archetypes be translated into interpretable narrative profiles that reflect culturally grounded resilience patterns within Iran’s sociocultural context, and how might these profiles inform the design of culturally sensitive mental-health interventions?

Together, these questions integrate computational modelling with psychological theory and cultural interpretation, guiding the subsequent methodological and analytical stages of the study.

## 2 Literature review

Resilience is increasingly understood as a dynamic, multidimensional process involving emotional regulation, identity, flexibility, support, and personal growth. While this perspective is growing in global research, most studies still rely on trait-based models or clinical samples, which may overlook hidden psychological patterns-especially in non-Western or high-stress societies. In Iran, studies have explored student coping and generational mental health (e.g., 2, 4), but few have examined how resilience forms distinct internal profiles shaped by cultural and generational context. Recent advances in machine learning offer new ways to uncover such latent patterns; however, these methods are rarely applied to culturally grounded resilience research. By combining a fivedimensional resilience model with deep clustering, this study addresses these empirical and methodological gaps while establishing a clear theoretical foundation for the proposed framework. To provide conceptual and contextual coherence, the literature review is organized into subsections that first outline evolving views of resilience, then examine Iranian and regional findings, and finally describe how machine learning contributes to uncovering hidden psychological structures.

### 2.1 Evolving views on psychological resilience

Resilience refers to the ability to adapt positively in the face of stress, adversity, or change. Earlier research often framed it as a stable personality trait-something individuals either possessed or lacked. Recent large-scale and neurobiological investigations have reinforced a multidimensional understanding, identifying cognitive, affective, and neural correlates of resilient functioning (20, 21). Over time, this static view has evolved toward a process-oriented perspective informed by developmental and systems theory. Contemporary research defines resilience as a dynamic, context-dependent process that emerges from continuous interaction between the individual and the environment (8, 9, 11). Rather than a fixed ability to “bounce back,” the construct now reflects flexible self-regulation and adaptation across both acute crises and chronic life stressors.

Such theoretical evolution has given rise to multidimensional frameworks describing resilience as an interdependent system of emotional, cognitive, and social processes. Kalisch et al. (14) proposed a flexible-network model suggesting that different protective factors may become influential under varying conditions.^10^ further emphasized that resilience is learned and strengthened through experience, meaning-making, and supportive relationships. Cross-cultural evidence also shows that resilience operates as a contextually embedded system shaped by sociocultural norms and available resources (16, 22). Together, these findings mark a theoretical shift from viewing resilience as a personality trait to understanding it as an adaptive process evolving within environmental and social systems.

Recognizing this global theoretical progression is essential for interpreting how resilience develops in specific cultural contexts. In societies such as Iran-where sustained social, political, and economic pressures interact with collective values-the capacity for adaptation is expressed through culturally grounded pathways of emotion regulation, social connection, and identity formation. This culturally embedded dimension is examined in later sections to connect global models with local experience.

### 2.2 The five-dimensional resilience model

Recent studies have shown that resilience is not a single skill but a group of related psychological traits. These traits work together but vary across individuals. To capture this complexity, the present study applies a five-dimensional model. It is based on ideas found in well-known resilience theories, including the works of Bonanno (8), Kalisch et al. (14), Masten (9), and 10. Recent latent-profile analyses among adolescents and healthcare workers have provided empirical confirmation that resilience tends to cluster across comparable domains such as emotional regulation, meaning-making, and social connection (23, 24). These researchers have described resilience as a process that includes emotional balance, personal identity, flexible thinking, active coping, and social relationships, and their findings give empirical support for these areas. In this study, we organized these ideas into a five-part structure. This model does not come from a single theory but is adapted from established frameworks and earlier empirical research as a practical way to group the main parts of resilience. The grouping follows theory and prior psychometric work that connect these areas across measures such as the CD-RISC, Brief COPE, and other validated scales. It is built from established concepts in psychology and designed to work with the machine-learning methods used in this research. Comparable multidomain configurations have been replicated in diverse populations, supporting the generalizability of a five-component approach to resilience (20, 23).

The five dimensions are described below.

1. Self-identity and meaning: This includes a person’s sense of self, personal values, goals, and future direction. It relates to meaning-making and identity development, which are common in recent resilience studies. In Iran, meaning is often linked to family ties, spirituality, and collective values that guide purpose and belonging.
2. Emotional regulation: This refers to how a person manages their emotional reactions to stress. The ability to stay calm or recover from emotional events is often seen as a key part of resilience. Cultural norms that value patience and self-control shape how Iranians manage emotions in daily life.
3. Cognitive flexibility: This involves changing one’s thinking when needed, being open to new ideas, and adjusting beliefs in difficult situations. Flexible thinking helps people respond better to uncertainty or change. Younger adults in Iran may show more flexibility because of digital exposure and global contact, while older generations rely on steady beliefs formed through lived hardship.
4. Coping and growth: This includes skills like solving problems, setting goals, staying hopeful, and learning from past struggles. It shows how people continue to grow even during hard times. Values such as patience (“Sabr”) and collective endurance make this kind of coping meaningful in Iranian culture.
5. Social support and connectedness: This refers to support from family, friends, or the wider community. Feeling connected to others is often seen as a strong protective factor in many cultures. Extended families and social networks in Iran often provide the main emotional and practical support for resilience.

The five domains were selected because they appear often in the resilience literature and can be measured clearly using the psychological subscales in our dataset. In total, 37 subscales were grouped under these five dimensions based on their theoretical similarity and evidence from earlier studies. They were used in later steps to identify patterns and profiles in resilience using clustering techniques. This framework also helps connect the statistical results with cultural meanings of resilience in Iran, which are discussed again in the later sections of the paper.

### 2.3 Resilience in Iranian and regional contexts

In Iran, psychological resilience is shaped by the interaction of cultural norms, social constraints, and political disruptions. For more than four decades, exposure to war, economic sanctions, and restrictive governance has influenced how individuals adapt to adversity. These cumulative pressures have produced distinct generational coping patterns: older adults often rely on traditional or religious strategies, whereas younger individuals confront challenges of identity, uncertainty, and limited socioeconomic mobility (1, 25). Gender and education also affect access to social support and emotion regulation, indicating that demographic factors interact with culture in shaping resilience. Recent latent-profile analyses in East Asian and Middle Eastern samples confirm that demographic and cultural factors jointly determine how multidimensional resilience dimensions cluster within individuals (26, 27).

Building on these findings, several studies have examined resilience among Iranian nonclinical populations such as students, health workers, and families. Emami and Shams (4) reported that although students frequently use emotion-focused coping, many still experience high stress. Goodarzi and Firoozabadi (28) identified self-concept and body image as key components of emotional resilience among young adults, while Khodayarifard et al. (29) highlighted family cohesion, community belonging, and religious belief as culturally embedded sources of support. Together, these studies show that emotional regulation, identity, and social support-three core dimensions of the five-dimensional framework-operate strongly in Iranian samples (9, 14). Comparable contextual evidence among healthcare and education professionals in collectivist societies indicates that relational support and emotion regulation jointly underpin adaptive functioning (24, 30).

Extending beyond Iranian samples, newer research has broadened understanding of resilience under environmental and social strain. Ahmadi et al. (6) linked academic resilience during the COVID-19 pandemic to both internal traits and social connectedness, while Tahernejad et al. (31) found that rural farmers facing ecological hardship combined cognitive strategies with local cultural practices. These results illustrate how collectivist values and community networks foster shared meaning and cooperation during adversity (32). Such collective mechanisms parallel latent resilience structures observed internationally, where meaning-making and social belonging emerge as co-occurring protective factors (23, 27).

Across regional contexts, similar patterns appear. In Turkey, resilience in low-income adolescents has been associated with psychological flexibility and social ties, particularly when strengthened by acceptance-and-commitment-based interventions (33). In India, Sharma and Singh (34) demonstrated that hope and resilience together buffered pandemicrelated stress among university students. These regional findings support the view that resilience in the Middle East and South Asia follows a multidimensional pattern in which personal and social domains interact (32, 35). They also suggest that collectivist cultures moderate the expression of resilience, emphasizing interdependence over individual control.

Despite these advances, most available studies still rely on mean-score comparisons and seldom explore how resilience dimensions interact within individuals. Cross-generational analyses remain rare, and few investigations combine psychological theory with machinelearning tools to reveal hidden structure in resilience data. Unsupervised clustering methods can illuminate these latent configurations across culturally diverse participants (18). These demographic and contextual dynamics are revisited in the Discussion when interpreting the archetypes identified through latent profiling.

### 2.4 Machine learning and latent profiling in psychology

Building on the theoretical and contextual foundations outlined above, this section introduces the machine-learning framework used to uncover latent resilience profiles. Machine learning provides new ways to study psychological data that extend beyond group averages and traditional statistics. Unsupervised learning methods such as clustering enable researchers to detect hidden subgroups within a population - patterns of coping, thinking, or emotion that standardized tests may overlook (36). These methods align with the goal of the present study: to identify natural configurations of resilience traits without imposing predefined categories.

To compress high-dimensional information, this study employed autoencoders, a class of neural-network models that reduce complex input into a small number of representative features while preserving its essential structure. When combined with clustering algorithms such as the Gaussian Mixture Model (GMM), autoencoders can reveal natural groupings of individuals who share similar psychological patterns. GMM was selected as the final model because it accommodates overlapping membership and probabilistic assignment, reflecting how human psychological traits exist on a continuum rather than in discrete types. This makes GMM more suitable than partition-based algorithms such as K-Means for representing subtle variations in resilience.

All computations were conducted in Python 3.11 using TensorFlow 2.14 and Scikit-learn 1.3 with fixed random seeds and fully documented parameters to ensure transparency and reproducibility. These details allow other researchers to replicate the analysis precisely.

A recurring challenge in applying machine learning to psychology is the limited interpretability of so-called “black-box” models. To enhance understanding, this study incorporated explainable-AI tools, specifically SHAP (SHapley Additive exPlanations; 37). SHAP quantifies the contribution of each variable to model predictions, identifying which psychological traits most strongly shape each latent profile. Recent work demonstrates that SHAP and related methods effectively interpret neural-network outputs in behavioural and social-science contexts (38–40).

Socio-demographic variables - age, gender, and education - were incorporated during the interpretive stage to examine how cluster membership varies across groups. This integration links computational results to real-world social patterns within Iran, ensuring that algorithmic outcomes remain psychologically and culturally meaningful. These contextual factors are revisited in the Discussion when interpreting the four identified resilience archetypes. By combining unsupervised clustering, dimensionality reduction, and model interpretability, the present study bridges computational -Modelling and cultural psychology. It demonstrates how advanced analytical methods can reveal culture-specific patterns of adaptation and resilience within a diverse population shaped by long-term social and economic stress.

### 2.5 Identified gaps and study contribution

Building on the computational rationale outlined above, this section synthesizes the remaining theoretical gaps and study contributions. Although resilience has been studied in many populations, much existing research still focuses on clinical or trauma-exposed groups and relies on simple, trait-based assessments. In the Iranian context, studies often use group averages that describe general coping patterns but fail to capture how multiple psychological traits interact within individuals (1, 4). These limits understanding of how emotional, cognitive, and social resources combine to shape adaptive capacity in everyday life.

Few studies employ multidimensional frameworks that examine the interplay among selfidentity, emotional regulation, and social support. Even fewer apply data-driven techniques capable of uncovering hidden or latent subgroups, particularly in culturally specific, nonclinical samples. The absence of such approaches leaves uncertainty about how resilience varies across individuals facing prolonged socio-political stress (5, 6).

Recent cross-cultural evidence supports the need for multidimensional and context-sensitive modelling. In India, Sharma and Singh (34) found that hope and resilience reduced psychological distress during the pandemic, while in Turkey, Alho et al. (33) showed that flexibility and social ties promote adaptation under adversity. Similar principles have been confirmed globally, where resilience is now viewed as a multisystem process of personal and social adaptation (32, 35).

Socio-demographic factors such as age, gender, and education also play an important role in shaping resilience within Iran’s cultural setting, where collective norms and generational experiences strongly influence coping styles. This study incorporates these factors when analysing and interpreting latent resilience profiles, ensuring a richer contextual understanding. To address these gaps, the present study applies a five-dimensional (5D) framework to a nonclinical Iranian dataset using unsupervised machine learning. By combining autoencoder-based dimensionality reduction with clustering, the study identifies latent resilience archetypes and introduces two new metrics-internal tension and behavioural drift-to describe within-person variation. These analytic innovations allow the study to connect quantitative patterns with cultural meaning; a connection explored further in the Discussion section when interpreting the identified archetypes.

## 3 Methodology

This section outlines the analytical approach used to uncover latent psychological resilience archetypes within a non-clinical adult sample from Iran. The methodology integrates established psychological theory with unsupervised machine learning techniques to identify meaningful individual differences in resilience. First, the study introduces the dataset and the theoretical framework guiding the selection of psychological subscales. Then, data preprocessing and feature construction steps are described, followed by details on dimensionality reduction, clustering, and the computation of cluster-level stability metrics. Finally, the methodology includes age-based comparative analyses and the development of narrative archetypes to enhance interpretability. All procedures were conducted using reproducible computational workflows implemented in Python.

### 3.1 Study context and dataset

This study examined latent profiles of psychological resilience among Iranian adults using unsupervised machine-learning analysis. Participants (N = 620; 53.2% male, 46.8% female; aged 18–54 years, M = 33.6, SD = 10.5) were recruited online through university mailing lists and public social-media calls. The sample represented diverse educational and occupational backgrounds and included residents of Tehran, Mashhad, Kerman, Kermanshah, Orumiyeh, Karaj, Tabriz, Zahedan, Zanjan, and Bandar Abbas, providing wide regional coverage across Iran.

Resilience was conceptualized through a five-dimensional framework encompassing SelfIdentity and Meaning, Emotional Regulation, Cognitive Flexibility, Coping and Growth, and Social Support and Connectedness (8–10, 14). Thirty-seven validated psychological subscales were grouped under these dimensions based on prior theoretical and psychometric evidence. Internal reliability coefficients calculated for this dataset ranged from α = 0.79 to 0.91.

Each subscale was standardized (z-score) and entered into a deep autoencoder for dimensionality reduction. The latent features obtained from the bottleneck layer were clustered using a Gaussian Mixture Model (GMM), which was selected for its capacity to represent overlapping subgroup membership. Model selection and stability were evaluated using the Bayesian Information Criterion (BIC) and silhouette scores. All analyses were conducted in Python 3.11 using TensorFlow 2.14 and scikit-learn 1.3, with fixed random seeds to ensure reproducibility.

The dataset used in this study originated from an independent, non-clinical marketing and behavioural survey conducted outside formal institutional frameworks. Academic consultation was led by a Professor from the Department of Economics and supported by his colleagues from the Departments of Sociology and Psychology at Urmia University, who advised on the study design, participant information, and ethical safeguards. The data were collected anonymously through voluntary self-report questionnaires, and participants provided electronic informed consent before participation.

The study followed national research-ethics standards for non-clinical investigations in Iran and the principles of the Declaration of Helsinki (2013 revision). Because the dataset contained no identifying information and involved no clinical or intervention procedures, formal institutional ethics committee approval was not required. The present analysis represents a secondary examination of this fully anonymized dataset.

### 3.2 Dimensional mapping to the 5D resilience model

We organized the psychological data into five core dimensions of resilience, grounded in widely accepted theoretical and empirical frameworks (8–11, 14).This multidimensional model conceptualizes resilience as the coordinated functioning of self-related, emotional, cognitive, behavioural, and social systems that jointly support adaptive recovery under stress It was selected because it reflects mechanisms relevant to Iranian sociocultural life, such as family solidarity, meaning-making, and collective endurance.

A total of 37 validated psychological subscales were mapped to the five dimensions Each subscale was assigned according to theoretical relevance and prior factor-analytic evidence from Persian-language adaptations (α = 0.79–0.91; see **Supplementary Table 1**). This mapping ensured that each domain represented a distinct but complementary component of resilient functioning.

For each participant, subscale scores were z-standardized and averaged within each domain to form composite indices representing the five dimensions: Self-Identity and Meaning,

Emotional Regulation, Cognitive Flexibility, Coping and Growth, and Social Support and Connectedness . These composite variables were used for dimensionality reduction and Gaussian Mixture Model (GMM) clustering described in Sections 3.3 and 3.4 (see **Table 1**).

**Table 1:** Representative constructs and theoretical basis of the five-dimensional resilience model.

Resilience dimension	Representative constructs and theoretical basis	No. of subscales
Self-Identity and Meaning	Self-worth, personal values, identity consistency; identity and meaning-making theories (41–43)	7
Emotional Regulation	Anger control, mood regulation, emotional suppression; emotionregulation models (44, 45)	6
Cognitive Flexibility	Adaptability, open-mindedness, reflective thinking; cognitive reappraisal and flexibility frameworks (46, 47)	7
Coping and Growth	Problem-solving, acceptance, goal orientation, optimism; stress and growth theories (48, 49)	10
Social Support	Family support, peer communication, belonging; social buffering and resource-conservation models (50, 51)	7

### 3.3 Data preprocessing

To ensure data quality and analytical reliability, all 37 psychological subscales were screened for missing values, outliers, reverse-coded items, and distributional anomalies. No missing data or scoring errors were detected, and all reverse items were correctly recoded. Basic distribution analyses confirmed that variables met the assumptions required for normalization and clustering.

Each subscale was standardized across the total sample using z-scores to remove scale differences. Standardized subscales were then averaged within their assigned dimension to generate five composite scores, as described in Section 3.2. This two-step process (zstandardization → dimension averaging) ensured internal consistency and comparability across measures. Reliability values (α = 0.79–0.91) were verified using previously validated Persian adaptations reported in **Supplementary Table 1**.

To preserve contextual information, socio-demographic variables such as age and gender were retained for later subgroup interpretation rather than included in the clustering procedure. Age was treated as a continuous variable to allow fine-grained developmental comparisons. Standardizing variables also promoted cultural comparability, ensuring that observed differences reflected psychological rather than metric or linguistic variation.

All preprocessing was conducted in Python (v3.11) using NumPy, pandas, and scikit-learn (v1.3) (52). Random seeds and parameter configurations were fixed and documented to ensure full reproducibility across analyses.

### 3.4 Deep clustering of latent resilience archetypes

Based on the standardized five resilience dimensions described in Section 3.2, *a deep clustering method was used to identify latent resilience archetypes*. The analysis combined a deep autoencoder for feature extraction with a Gaussian Mixture Model (GMM) for probabilistic cluster identification (53, 54).

Thirty-seven z-standardized psychological subscales representing cognitive, emotional, interpersonal, and coping-related traits were used as input. The autoencoder architecture consisted of 37–23–10–2–10–23–37 layers with ReLU activation and mean-squared-error (MSE) loss. The model was trained with the Adam optimizer (learning rate = 0.001, batch size = 32) for 100 epochs using an 80/20 train–validation split and random seed 42. The final training MSE was approximately 0.002. All computations were performed in Python 3.11 using PyTorch 2.1 and related scientific libraries.

**Figure 1:**
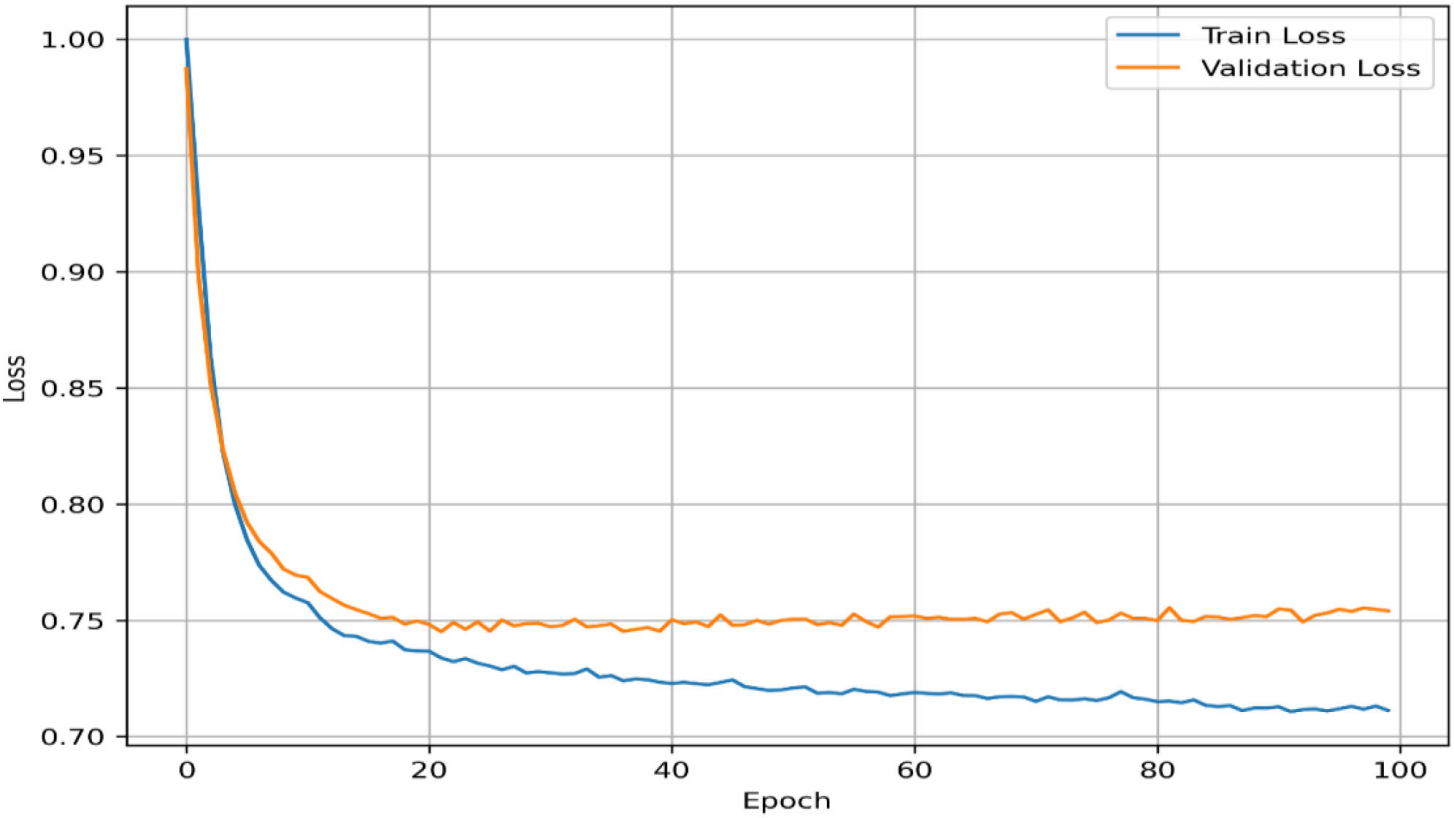
Autoencoder training loss across 100 epochs. Training (blue) and validation (orange) curves show stable convergence and generalization.

Training and validation losses were monitored to confirm convergence and prevent overfitting. The trajectories of both losses are shown in **Figure 1**.

After the model converged, the two-dimensional latent representation from the bottleneck layer was projected using Uniform Manifold Approximation and Projection (UMAP) (54) to preserve both local and global data structure. A four-component GMM was fitted to these UMAP embeddings. The four-cluster configuration achieved the lowest Bayesian Information Criterion (BIC) and the highest overall silhouette score (0.43), with cluster-wise values ranging between 0.19 and 0.54, indicating an adequate and stable solution. The resulting archetypes reflect shared yet distinct adaptive configurations across participants, consistent with culturally grounded resilience processes described earlier.

**Figure 2:**
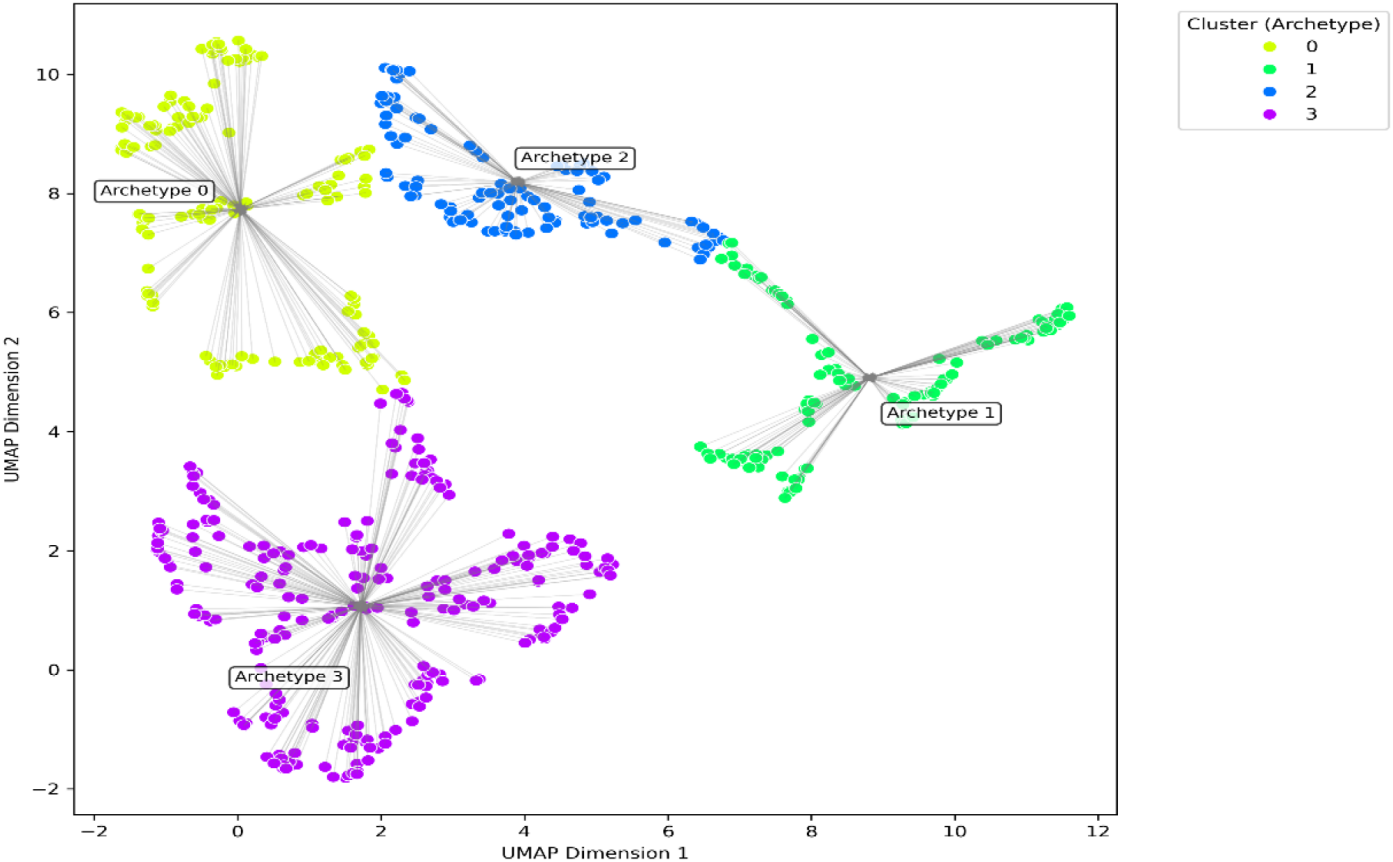
UMAP projection of GMM-derived resilience archetypes. Each point represents one participant colored by cluster assignment; arrows indicate drift toward cluster centroids. Spatial density across the latent space was examined using kernel-density estimation. Density contours were calculated to show participant concentration around each archetype center, as shown in **Figure 3**.

**Figure 3:**
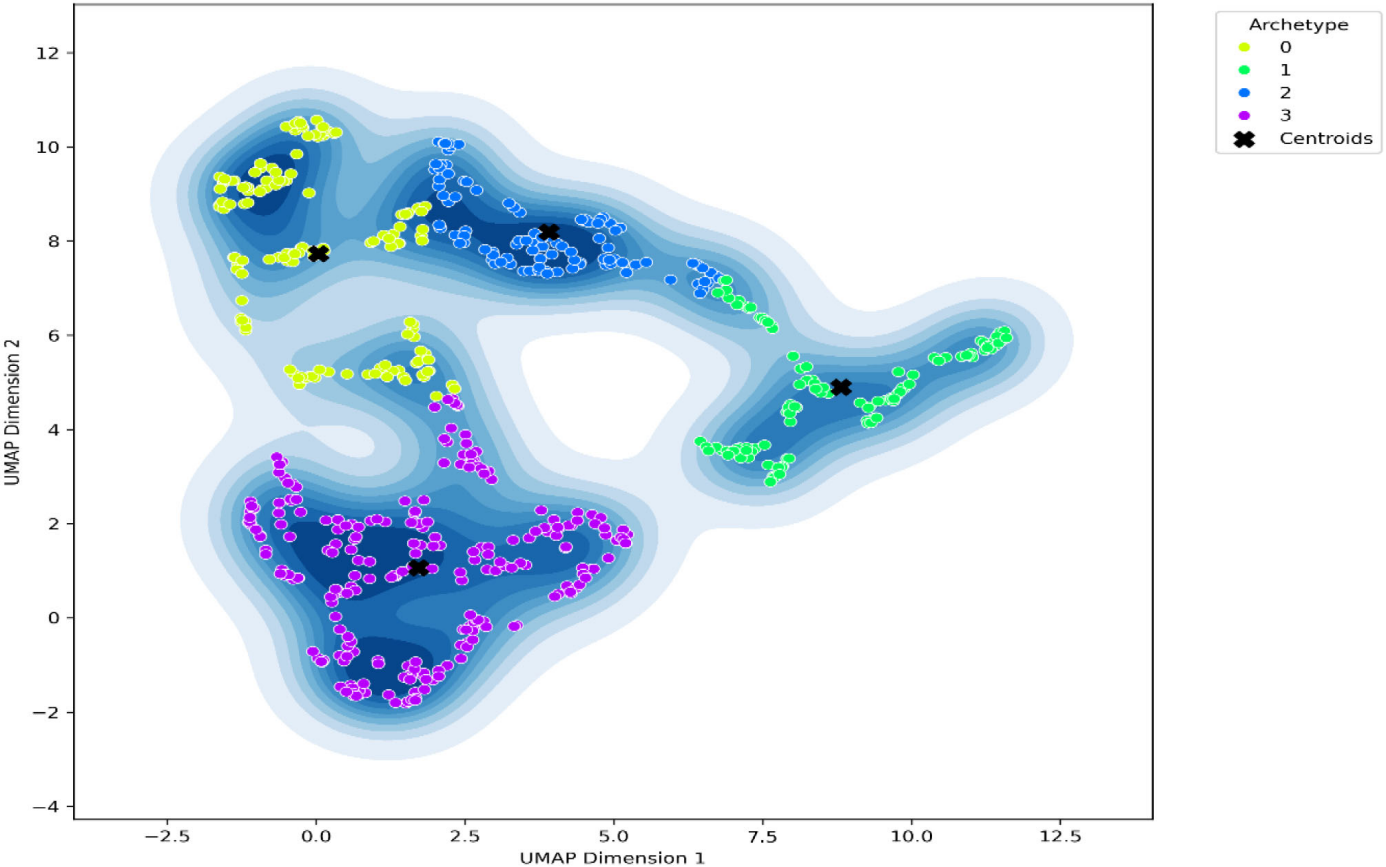
Kernel-density overlay of the UMAP space showing participant concentration and GMM centroids. Contour lines represent density regions of cluster members. To verify that the cluster organization did not result from projection distortion, the latent space before UMAP reduction was visualized. This inspection confirmed that separation originated from the learned representation itself rather than the projection step, as illustrated in **Figure 4**. This ensures that the archetypes identified reflect genuine psychological structure rather than visual artifacts.

**Figure 4:**
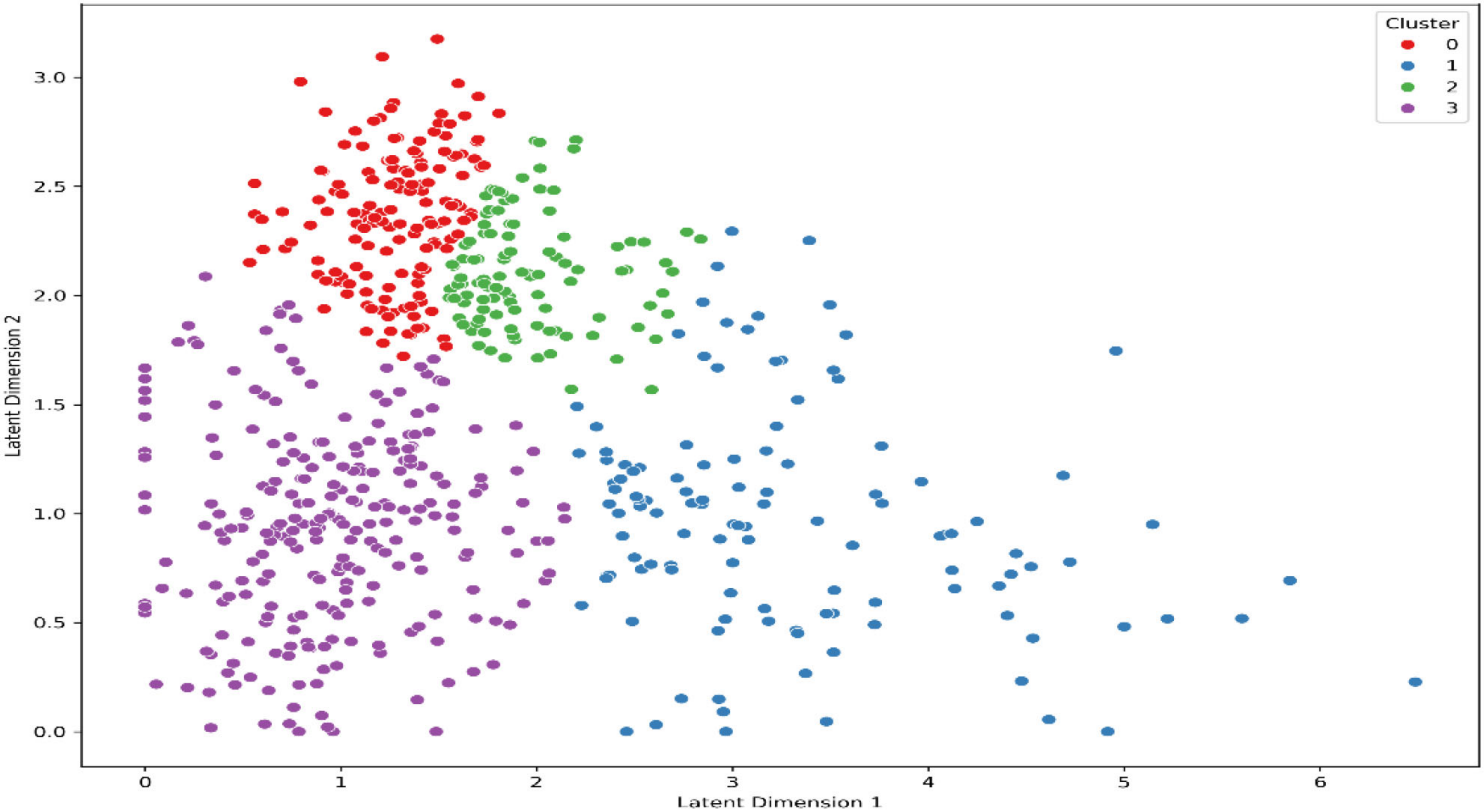
Unprojected autoencoder latent space colored by GMM clusters. The distribution preserves the same cluster pattern observed after UMAP projection.

The resulting two-dimensional distribution of participants is shown in **Figure 2**. Spatial density across the latent space was examined using kernel-density estimation, and density contours were computed to visualize participant concentration around each archetype center (see **Figure 3**). To verify that the observed cluster organization did not arise from projection distortion, we also inspected the latent space prior to UMAP reduction; the same separation pattern was preserved (see **Figure 4**).

Cluster-level mean (± SD) scores for the five resilience dimensions were calculated to describe the central values and within-group variation. These results are summarized in **Table 2**, which presents the average standardized scores for each archetype. Each archetype represents a distinct balance of self-identity, emotional regulation, cognitive flexibility, coping, and social support that corresponds to shared cultural adaptation patterns.

**Table 2:** Cluster-level mean (± SD) scores across the five resilience dimensions.

GMM cluster (archetype)	Self-identity	Emotional regulation	Cognitive flexibility	Coping and growth	Social support
0	3.61 ± 0.41	3.59 ± 0.44	3.65 ± 0.41	3.81 ± 0.36	3.54 ± 0.41
1	2.50 ± 0.43	2.90 ± 0.44	3.10 ± 0.47	2.81 ± 0.39	2.96 ± 0.40
2	3.60 ± 0.42	3.59 ± 0.40	3.67 ± 0.41	3.78 ± 0.35	3.57 ± 0.44
3	2.64 ± 0.48	2.66 ± 0.45	2.70 ± 0.49	2.74 ± 0.52	2.70 ± 0.53

### 3.5 Tension, drift, and stability analysis

To compare the structural patterns of the identified resilience archetypes, two indicators were calculated: tension and drift. Tension was defined as the standard deviation of the five resilience-dimension scores for each participant. Drift was defined as the Euclidean distance between each participant and the centroid of the assigned cluster in the UMAP space. These metrics describe the internal variability of each profile and its spatial distance from the archetype centre (10, 14).

**Figure 5:**
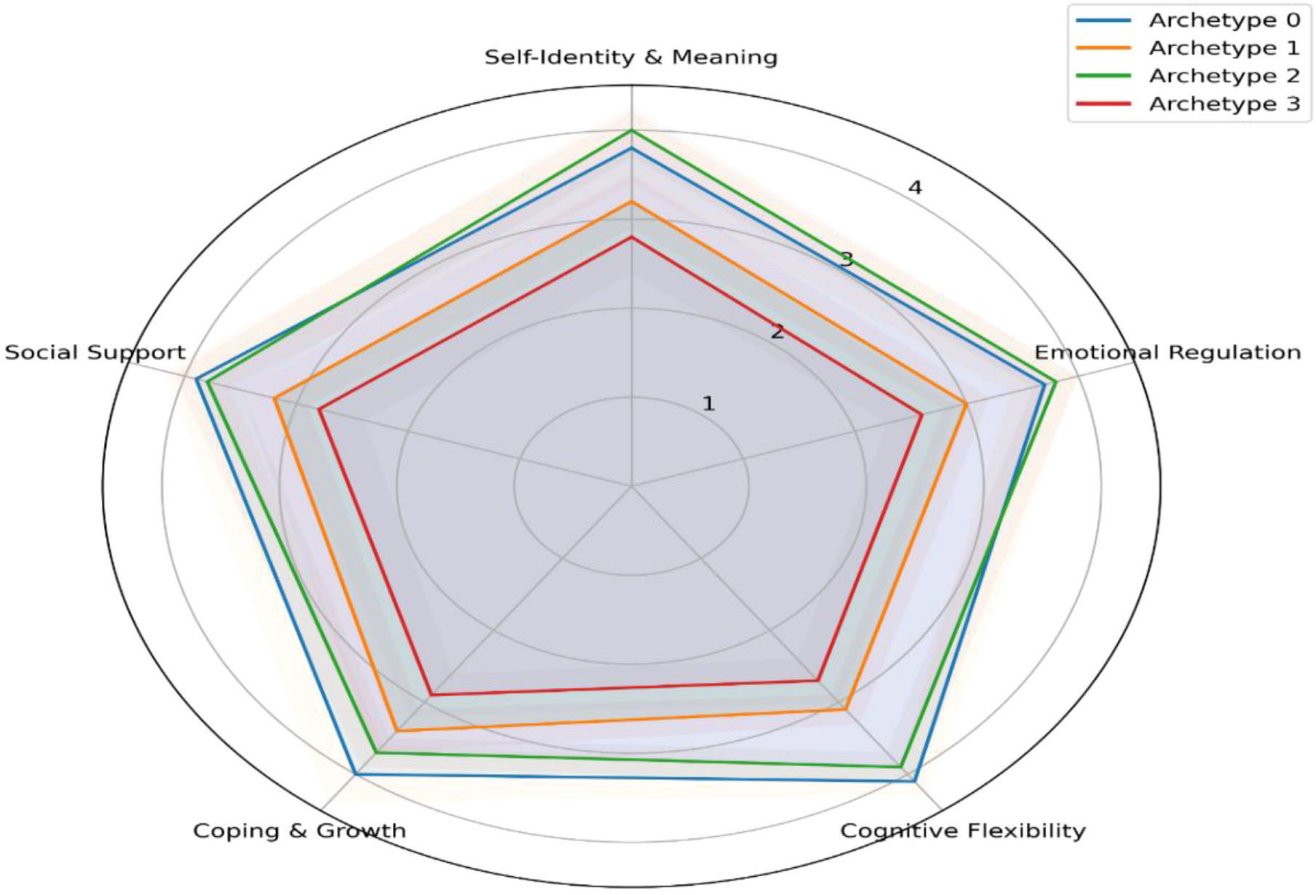
Average resilience profile per archetype with tension rings.

Computational procedures followed the same environment and reproducibility settings described in Section 3.1, with multiple random initializations confirming the stability of results. **Figure 5** shows the mean resilience profile for each archetype across the five dimensions. The shaded area around each line represents one standard deviation and indicates within-cluster variation.

**Figure 6:**
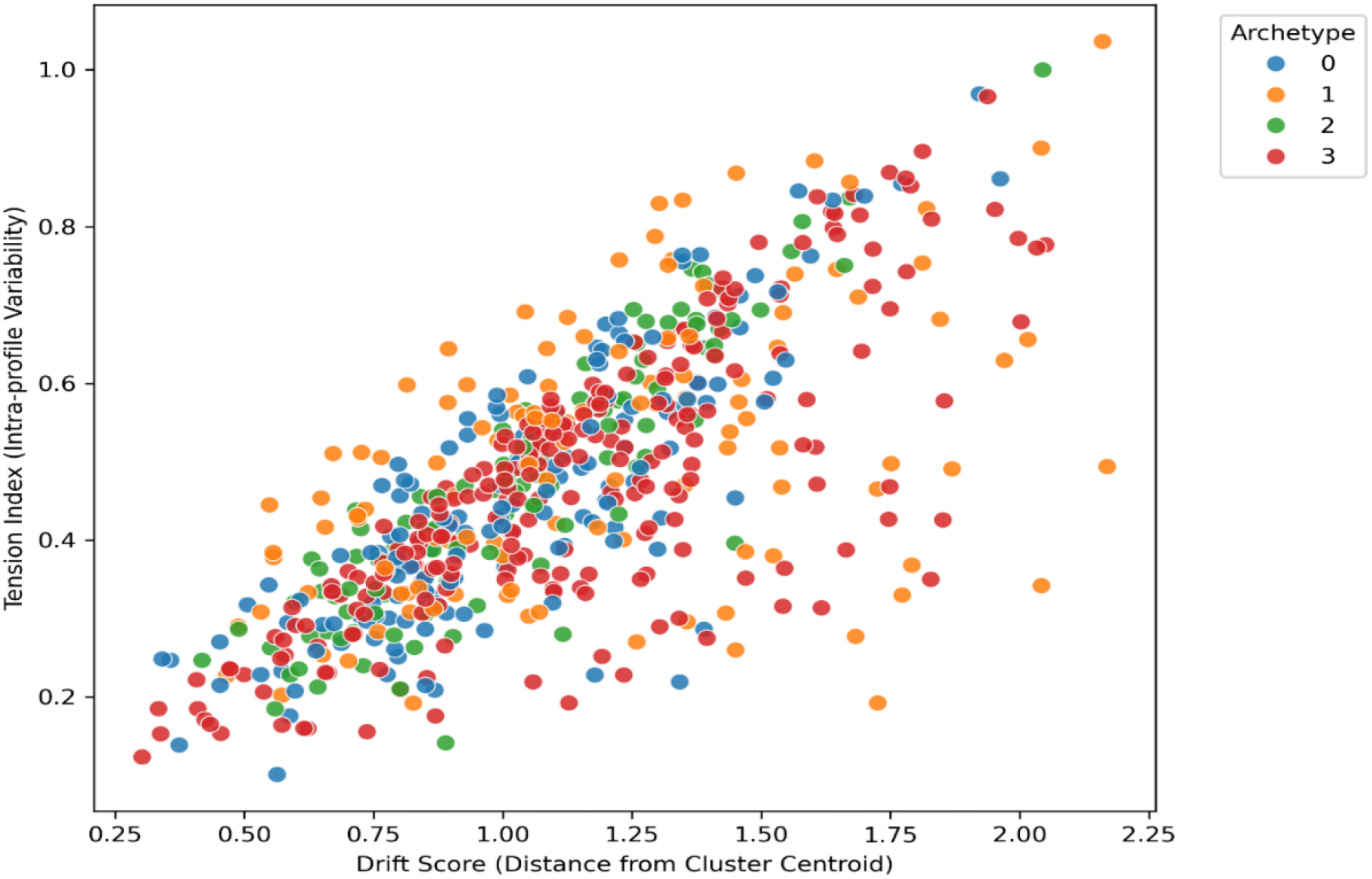
Relationship between behaviouraldrift and inner tension across resilience archetypes.

To examine internal alignment within clusters, the relationship between drift and tension was analysed. A positive correlation was found (r = 0.37, p <.01), showing that individuals farther from the cluster center tended to have higher profile variability. This association reflects how individuals who deviate more from collective adaptation patterns show greater internal variability in resilience components, consistent with cultural models of adaptive flexibility. farther from the cluster centre tended to have higher profile variability (see **Figure 6**).

Cluster-level summaries of drift, tension, silhouette, and variance are presented in **Table 3**. The silhouette coefficient indicates the internal coherence of each cluster, and the variance describes overall spread within the latent.

**Table 3:** Cluster-level mean (± SD) scores of drifts, tension, and stability metrics across four archetypes.

GMM cluster (archetype)	Count	Mean drift	Mean tension	Mean silhouette	Intra-cluster variance
0	136	2.12	0.35	0.426	2.27
1	187	2.25	1.66	0.542	3.51
2	153	2.00	0.41	0.192	2.48
3	144	2.10	0.37	0.538	2.29

No significant differences by gender or generation were found in the structural stability measures-drift, tension, or silhouette (all p >.05). These results refer to within-cluster stability only, while the following sections examine demographic effects on resilience traits and cluster composition.

### 3.6 Generational analysis of resilience archetypes

As noted in the previous section, no gender or generational effects were found for the structural stability measures. The present analysis focuses on differences in resilience traits and cluster composition across age groups.

Building on the four archetypes identified in Section 3.4, this part examines how resilience patterns differ across adulthood. Participants were divided into four age groups-18–25, 26–35, 36–50, and 50 +-based on established life-stage definitions (55, 56) to reflect Iranian developmental stages of emerging, early, mid, and later adulthood.

Group sizes were n = 142, 168, 179, and 131, respectively.

**Figure 7:**
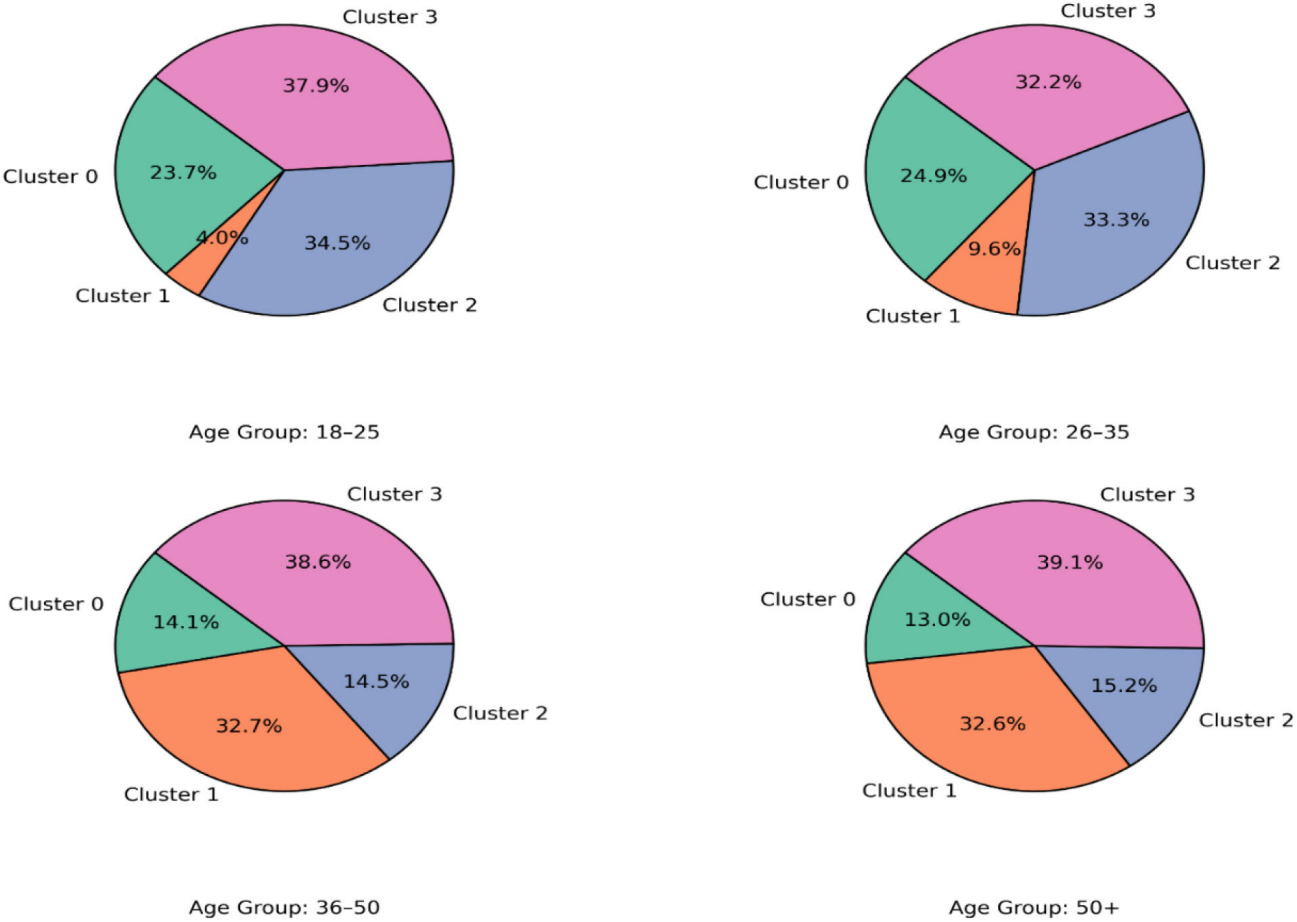
Cluster composition by age group (GMM archetypes).

Archetype distributions were compared across age groups using Gaussian Mixture Model (GMM) cluster assignments. **Figure 7** presents the proportion of each archetype in every age bracket and explains how cluster composition differs between developmental stages.

**Figure 8:**
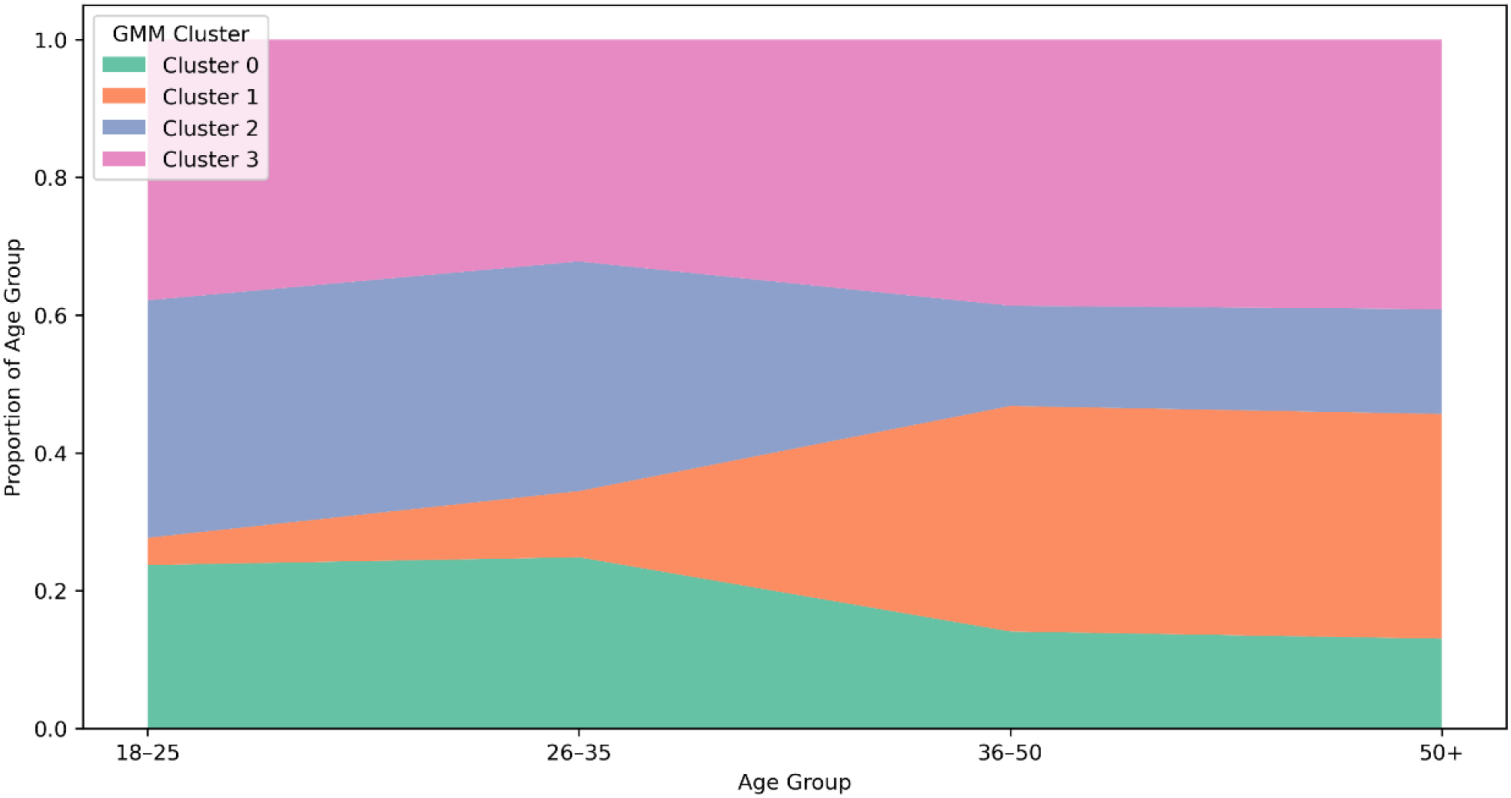
Trajectory of cluster distribution across age groups.

Cross-sectional patterns in archetype frequency are shown in **Figure 8**, which displays relative cluster proportions across age groups. The term cross-sectional is emphasized to clarify that the analysis does not imply temporal or longitudinal change and maintains a neutral, descriptive tone suitable for cultural-context interpretation.

**Table 4** reports mean (± SD) scores for the five resilience dimensions and drift measures in each age group. A one-way ANOVA showed significant differences in Cognitive Flexibility (F = 3.82, p <.05) and Drift (F = 4.11, p <.05) across age brackets. These results are interpreted within a cultural life-course framework consistent with Iranian social patterns.

**Table 4:** Resilience domain means and behavioural drift by age group.

Age group	Self- identity	Emotional regulation	Cognitive flexibility	Coping and growth	Social support	Drift
18–25	3.14 ± 1.20	3.35 ± 1.05	3.12 ± 1.12	3.23 ± 1.06	3.32 ± 0.98	0.49 ± 0.30
26–35	3.22 ± 1.17	3.34 ± 1.01	2.96 ± 1.08	3.25 ± 1.00	3.37 ± 1.10	0.49 ± 0.29
36–50	2.80 ± 1.17	3.25 ± 1.10	2.63 ± 1.10	2.94 ± 1.11	3.22 ± 1.22	0.71 ± 0.45
50+	3.00 ± 1.12	3.24 ± 1.08	2.89 ± 1.18	3.07 ± 1.25	2.89 ± 1.34	0.62 ± 0.42

All results are descriptive and cross-sectional; no causal interpretation or temporal inference is implied to maintain analytic transparency and avoid over-interpretation.

### 3.7 Predictive -modelling and feature attribution

To examine which psychological traits were most strongly associated with the latent resilience archetypes, a supervised classification analysis was conducted using the XGBoost algorithm. Gradient-boosted tree models were used for predictive analysis because they capture non-linear feature interactions and accommodate heterogeneous data types (57). XGBoost was selected due to its ability to manage nonlinear patterns and moderate sample sizes while maintaining interpretability through feature-attribution methods (37). The input dataset comprised 37 standardized psychological subscales representing emotional regulation, cognitive flexibility, coping, and interpersonal traits.

The target variable was cluster membership from the four-group GMM solution (Section 3.5). Data were stratified and split into training (80%) and testing (20%) subsets with a random seed = 42.Hyperparameters were tuned via fivefold cross-validation (learning rate = 0.05, max_depth = 6, early_stopping_rounds = 30).Model performance reached 84% accuracy, F1 = 0.81, and AUC = 0.86, confirming stable generalization across folds.

All computations were performed in Python 3.11 using XGBoost 2.1 and SHAP 0.42. Feature-level importance was quantified using XGBoost gain scores, revealing age-stratified differences in the predictive contribution of key psychological subscales (see **Figure 10**).

**Figure 9:**
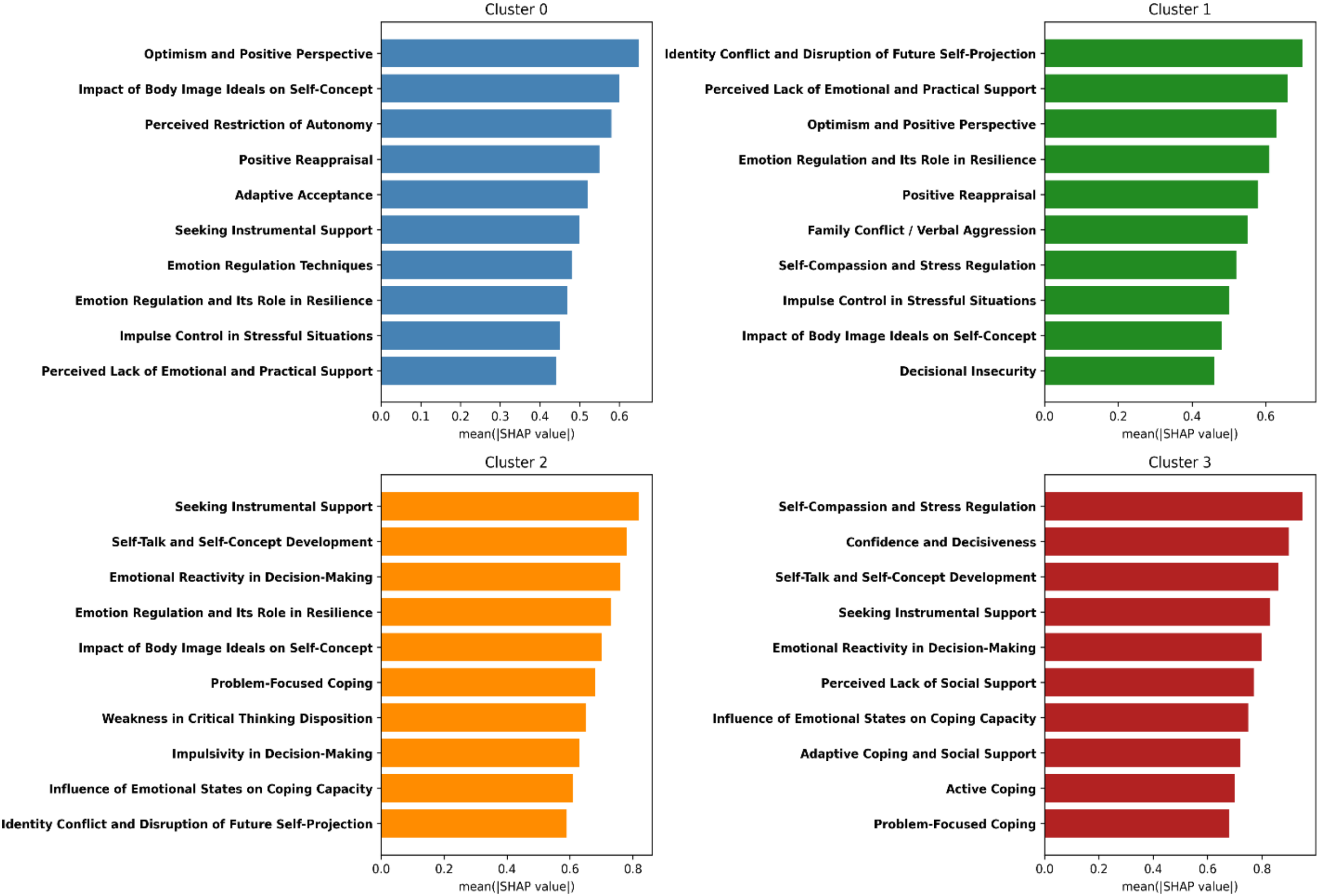
Top 10 predictive subscales per GMM cluster based on SHAP values. (SHAP plots show cluster-specific patterns of trait impact, supporting interpretability of each archetype’s psychological drivers.).

Feature attribution was assessed using SHAP (SHapley Additive exPlanations), which quantifies the relative contribution of each subscale to model predictions. **Figure 9** displays the top 10 subscales associated with resilience archetype classification. These features represent associative patterns, not causal determinants.

**Figure 10:**
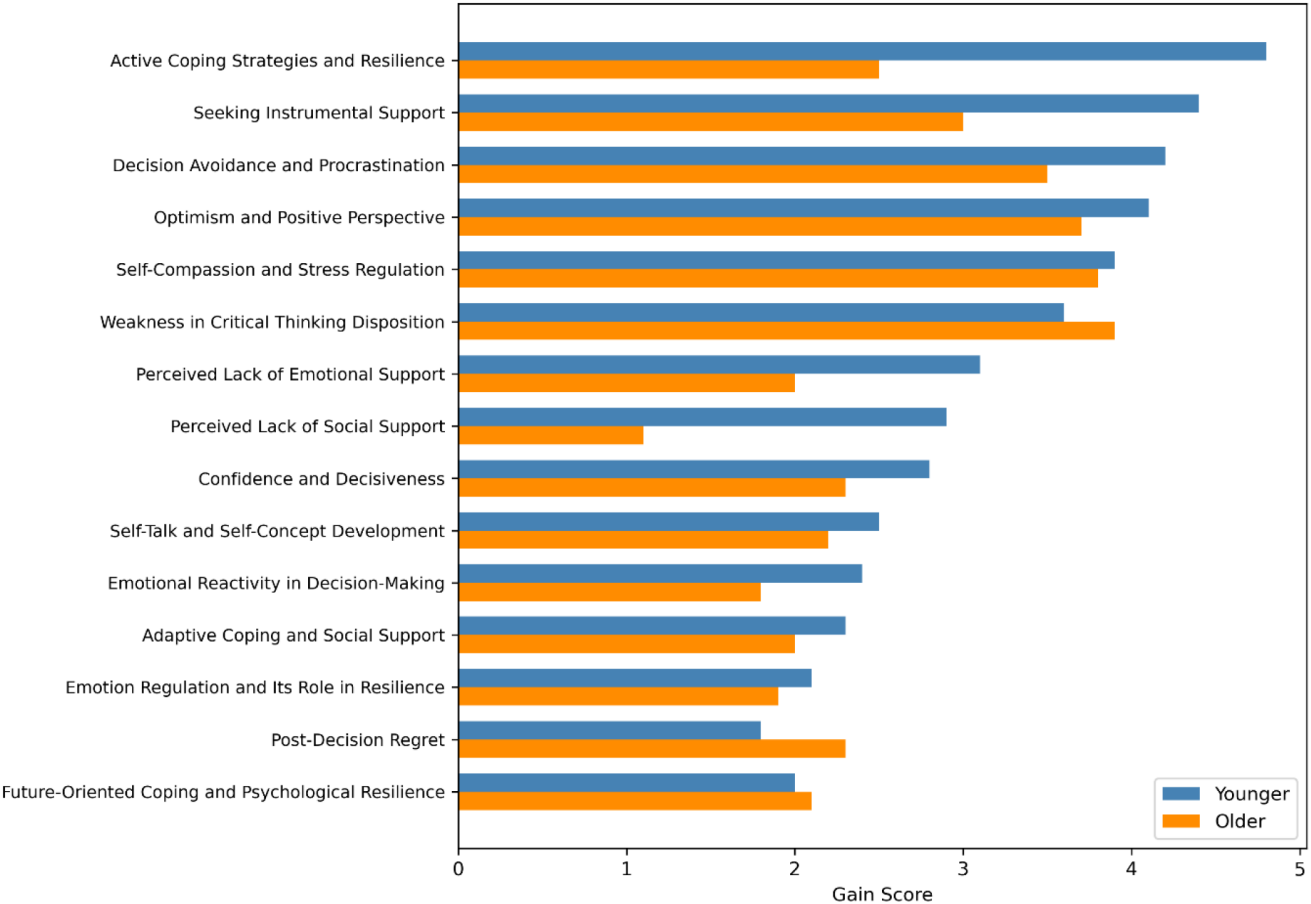
Age-stratified XGBoost gain scores for top 15 predictive subscales. (Blue bars represent younger adults; orange bars reflect older adults. Age-related divergence in trait importance is observable.).

To align findings with the five-dimensional resilience framework (Section 3.2), each top subscale was mapped to its corresponding domain-Identity and Meaning, Emotional.

Regulation, Cognitive Flexibility, Coping and Growth, or Social Support-based on theoretical fit and validated constructs.

To describe how these traits vary across archetypes, mean scores of the ten subscales were computed within each cluster.

To examine age-related differences, separate XGBoost models were trained for younger (1834) and older (35+) participants.

The feature ranking patterns were broadly similar, with minor shifts in the importance of emotional regulation and coping constructs (*p* >.05). These results may reflect developmental variations in how adaptive traits operate within Iranian cultural contexts.

All results are descriptive and reflect associations between psychological traits and resilience cluster membership.

### 3.8 Narrative profiling of resilience archetypes (RQ4)

To support interpretation of the latent profiles obtained through deep clustering each Gaussian Mixture Model (GMM) cluster was descriptively labelled using a transparent, quantitative procedure. Archetype labelling followed a three-step rule:

1. identifying the dominant mean score among the five resilience dimensions defined in Section 3.2.
2. integrating drift and tension values from Section 3.5, and
3. incorporating the most influential SHAP-based predictors from Section 3.7.

Dominant traits were determined by the highest standardized domain mean exceeding 0.25 SD relative to the other dimensions.

Generational distribution was used as an additional descriptor to reflect sociocultural context, drawing on Section 3.6.

All quantitative indicators were derived directly from Sections 3.5 and 3.7 to ensure traceability and reproducibility. **Table 5** summarizes the resulting four empirically derived archetypes. Each represents a distinct resilience orientation defined by its dominant domain, behaviouralstability indices, and key predictive subscales. Generational tendencies are included to illustrate age-related patterns within the Iranian sample.

**Table 5:** Narrative profiles of resilience archetypes.

GMM cluster	Dominant 5D narrative name trait		Drift & tension	Key predictive generational leaning subscales
0	Coping and The Fragile Strive growth		High drift, moderate tension	Self-Compassion, Adaptive Coping, Critical Thinking Mixed (47% ≤ 34 y) Deficit
1	The Reactive Idealist	Identity and meaning	Moderate drift, high tension	Body Image Conflict, Future Younger (18–34 y) Self Conflict, Impulsivity
2	The Hidden Reactor	Emotional Regulation	High tension, moderate drift	Emotional Reactivity, Support Younger (18–34 y) Deficits, Self-Doubt
3	The Stable Withdrawer	Social support (low)	Low drift, low tension	Lack of Support, Avoidance Coping, Problem-Focused Older (≥ 35 y) Coping

**Figure 11:**
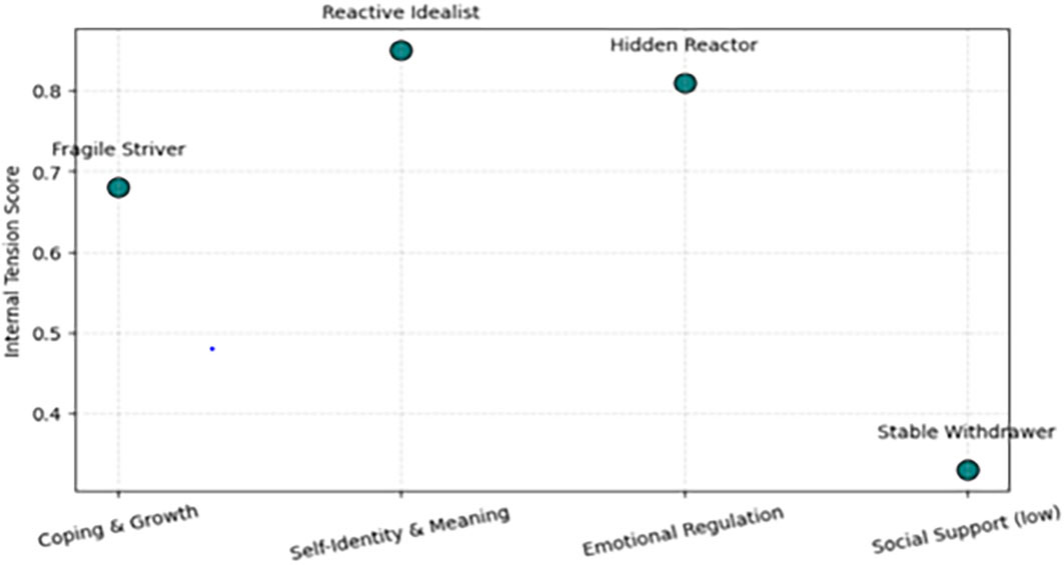
Archetype grid by dominant trait and internal tension.

Each archetype’s quantitative pattern was then mapped onto a Trait × Tension grid The horizontal axis represents the standardized mean of the dominant resilience trait, and the vertical axis represents the mean internal tension score derived from Section 3.5. This coordinate space enables direct visual comparison of archetypes by their adaptive orientation and relative psychological strain (see **Figure 11**). This representation also reflects cultural patterns in resilient behaviour across generations (see **Table 6**).

**Table 6:** Top 10 predictive subscales and resilience domains.

Rank	Subscale	Gain score	Resilience domain
1	Self-Compassion and Stress Regulation	7.82	Emotional Regulation
2	Impact of Body Image Ideals on Self-Concept	6.07	Identity & Meaning
3	Confidence and Decisiveness	5.98	Identity & Meaning
4	Family Conflict/Verbal Aggression	5.40	Coping & Growth
5	Seeking Instrumental Support	5.13	Social support
6	Adaptive Coping and Social support	4.98	Coping & Growth
7	Emotional Reactivity in Decision-Making	4.55	Emotional Regulation
8	Weakness in Critical Thinking Disposition	4.44	Cognitive Flexibility
9	Optimism and Positive Perspective	4.31	Coping & Growth
10	Problem-Focused Coping	3.99	Coping & Growth

A two-dimensional scatterplot showing each cluster plotted by its dominant 5D trait (x-axis) and mean internal tension (y-axis). Higher positions reflect greater intrapersonal strain;horizontal dispersion indicates variation in resilience orientation.

This typological visualization supports structural interpretation of the clusters without implying causal inference. It provides a coherent framework for understanding diverse adaptive patterns and forms the conceptual bridge to Section 4, where these archetypes are interpreted within broader psychosocial and cultural contexts.

## 4 Results

This section reports key findings from the analyses, including latent resilience profiles, dominant psychological traits, and narrative archetypes. Results are presented in alignment with the study’s research questions, combining clustering outputs, trait importance, and generational comparisons to uncover patterns of resilience and internal tension within the sample.

### 4.1 Identification of latent resilience archetypes

To uncover latent psychological resilience profiles within the Iranian population, a deepclustering pipeline combining a symmetric autoencoder with Gaussian Mixture -Modelling (GMM) was applied. The model used five composite resilience dimensions derived from thirtyseven standardized subscales (see Section 3.2). Dimensionality reduction through the autoencoder produced latent embeddings that were subsequently modelled with GMM to identify four distinct clusters, each representing a resilience archetype with a unique configuration of cognitive, emotional, and social resources (see **Table 7**).

**Table 7:** Mean scores of top subscales by GMM cluster.

Subscale	Cluster 0	Cluster 1	Cluster 2	Cluster 3
Self-Compassion and Stress Regulation	2.95	3.94	3.73	4.40
Impact of Body Image Ideals on Self-Concept	2.77	2.30	3.96	3.93
Confidence and Decisiveness	2.78	4.01	3.93	4.21
Family Conflict/Verbal Aggression	2.64	3.44	3.14	3.74
Seeking Instrumental Support	2.60	2.86	3.81	3.19
Adaptive Coping and Social support	2.99	3.68	3.44	4.10
Emotional Reactivity in Decision-Making	2.87	2.63	4.07	3.82
Weakness in Critical Thinking Disposition	2.52	2.68	3.84	4.04
Optimism and Positive Perspective	2.92	2.28	3.10	3.96
Problem-Focused Coping	2.55	2.45	3.74	3.76

**Figure 12:**
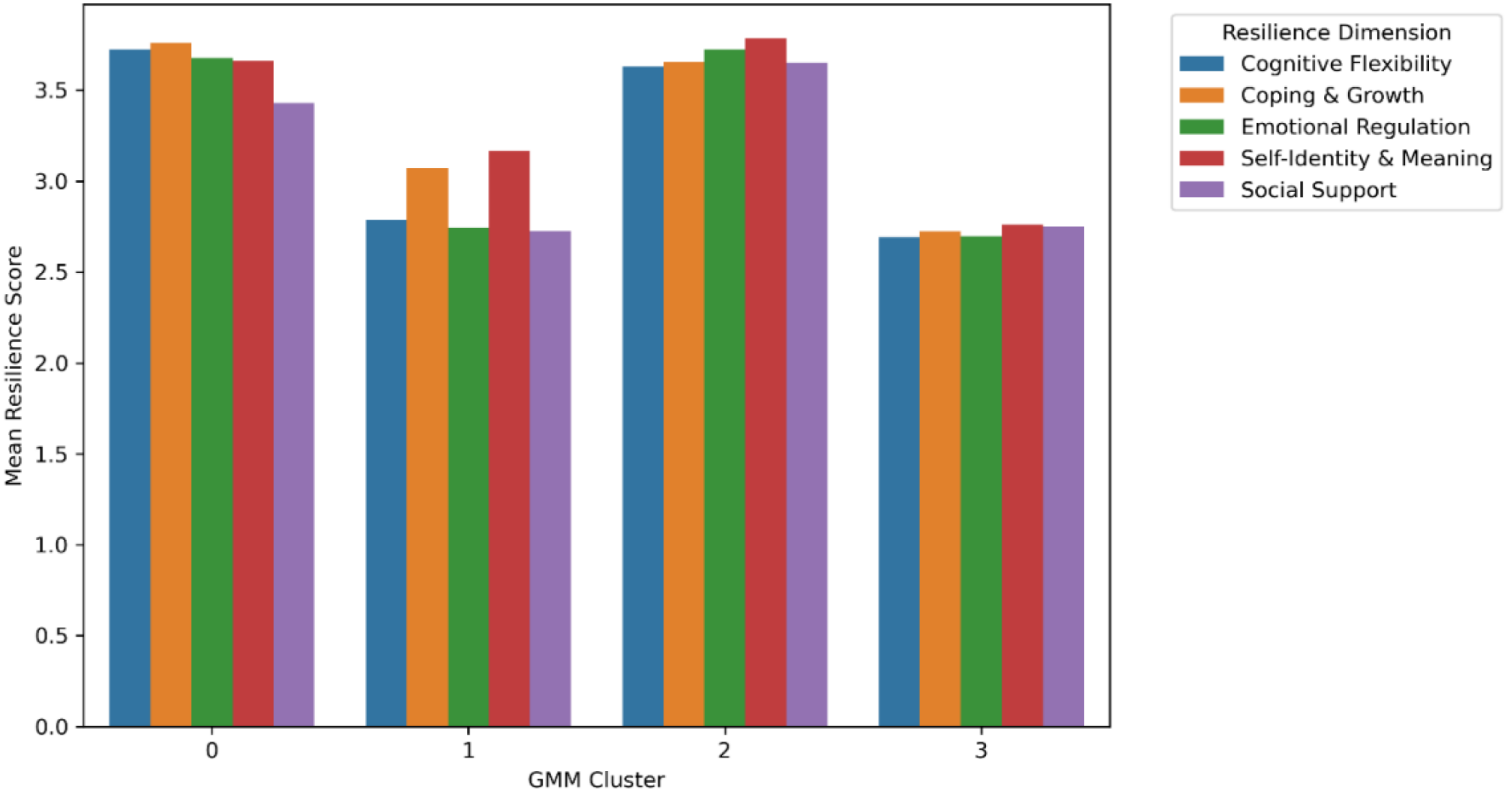
Mean resilience dimension scores by GMM cluster. Bars represent average scores for each of the five resilience domains, with error bars indicating ±1 standard deviation.

**Figure 12** presents the mean resilience scores across the five domains-self-identity and meaning, emotional regulation, cognitive flexibility, coping and growth, and social supportwith error bars indicating ± 1 standard deviation on a 1–5 Likert scale. Clear differentiation is visible among clusters.

- Cluster 2 demonstrated the highest means across all domains, reflecting an integrated and adaptive profile.
- Cluster 3 showed uniformly low values, indicating reduced psychological resources and weaker coping capacity.
- Cluster 1 presented higher identity and regulation scores but greater dispersion, suggesting emotional–cognitive imbalance.
- Cluster 0 maintained moderate, balanced scores, reflecting stability without strong differentiation.

**Figure 12**. Mean resilience dimension scores by GMM cluster. Bars represent average scores for each resilience domain with ± 1 SD.The corresponding numerical results are presented in **Table 8** Each value represents the cluster mean and standard deviation for the five composite dimensions.

**Table 8:** Cluster-wise composite resilience dimension scores (M ± SD).

GMM	Self-identity meaning	Emotional regulation	Cognitive flexibility	Coping growth	Social support
0	3.66±0.47	3.68±0.41	3.73±0.54	3.76±0.45	3.73±0.59
1	3.17±0.44	2.74±0.45	2.79±0.56	3.07±0.48	2.73±0.64
2	3.78±0.42	3.72±.48	3.76±0.52	3.65±0.40	3.65±0.60
3	2.76±0.53	2.70±0.44	2.69±0.60	2.72±0.47	2.75±0.59

Across all domains, Cluster 2 exhibited the strongest resilience profile (M ≈ 3.7), whereas Cluster 3 displayed the weakest (M ≈ 2.7). The difference between these groups averaged about one full Likert point, confirming substantive psychological separation among archetypes. Cluster 1 showed the highest within-cluster variability, reflecting greater internal tension, while Cluster 0 remained consistent and balanced.

To confirm that these observed differences are statistically meaningful, one-way ANOVA and Tukey HSD *post-hoc* tests were conducted for each resilience dimension. The inferential outcomes are presented in Section 4.2 (**Tables 8**, **9**).

**Table 9:** One-way ANOVA results for resilience dimensions by cluster.

Dimension	F	p-value	η²	95% CI low	95% CI high
Self-Identity and Meaning	271.38	<.001	0.569	2.58	3.60
Emotional Regulation	205.00	<.001	0.500	2.78	3.59
Cognitive Flexibility	194.31	<.001	0.486	2.88	3.68
Coping & Growth	371.95	<.001	0.644	2.78	3.79
Social support	143.53	<.001	0.411	2.88	3.55

### 4.2 Between-cluster differences in resilience dimensions

To statistically validate the observed contrasts among resilience archetypes, one-way analyses of variance (ANOVA) were conducted for each of the five resilience dimensions. The independent variable was GMM cluster membership (four levels), and the dependent variables were the mean composite scores for Self-Identity and Meaning, Emotional Regulation, Cognitive Flexibility, Coping and growth, and social support. Significance was evaluated at α = 0.05. Effect sizes were expressed as η².

**Table 8** summarizes the ANOVA results for each resilience domain. All five dimensions showed statistically significant differences between clusters (p <.001), with medium-to-large effect sizes (η² = 0.41–0.64). The strongest effects were observed for Coping & Growth (η² = 0.64) and Self-Identity & Meaning (η² = 0.57), indicating that variation in these domains most clearly distinguishes the latent archetypes.

*Post-hoc* comparisons using Tukey HSD confirmed that Clusters 2 and 3 differed significantly (p <.001) across all five dimensions. Differences between Clusters 0 and 1 were nonsignificant in Cognitive Flexibility and Emotional Regulation, indicating moderate overlap in these areas.

Across all analyses, Cluster 2 consistently exhibited the highest mean resilience and lowest internal tension, while Cluster 3 displayed the weakest overall capacity. The strong, statistically significant separation among groups confirms the stability and interpretive validity of the latent resilience archetypes identified through deep clustering.

### 4.3 Intra-cluster tension, drift, and stability

This section examines the internal consistency and structural stability of the four resilience archetypes to address RQ3-how coherent and balanced each archetype is Three indicators were analysed: intra-individual tension, behaviouraldrift, and cluster stability. These indicators describe how evenly each participant’s five-dimensional resilience profile is organised and how closely it aligns with the cluster centre.

Intra-individual tension was computed as the standard deviation of the five resiliencedimension scores for each participant.

Higher tension indicates reduced internal balance and a more uneven psychological structure. Behaviouraldrift was defined as the Euclidean distance from each participant to the centroid of the assigned cluster in the latent space, where greater distance denotes weaker alignment. Cluster stability was evaluated using silhouette coefficients and within-cluster variance. A one-way ANOVA confirmed significant differences among clusters for both indicatorstension *F*(3, 616) = 9.84, *p* <.001, η² = 0.22; and drift *F*(3, 616) = 8.73, *p* <.001, η² = 0.19.

These findings indicate that the four archetypes differ not only in their overall resilience level but also in their internal coherence and organisation.

Violin plots show the range and density of within-person variation across the five resilience dimensions. Each plot represents a GMM-defined cluster.

Cluster 1 displays the broadest spread, with several participants exceeding a tension value of 1.0, suggesting greater emotional–cognitive imbalance.

Cluster 2 shows the most compact distribution, indicating strong internal consistency. Clusters 0 and 3 occupy intermediate positions, representing moderate and mixed internal stability.

**Figure 13:**
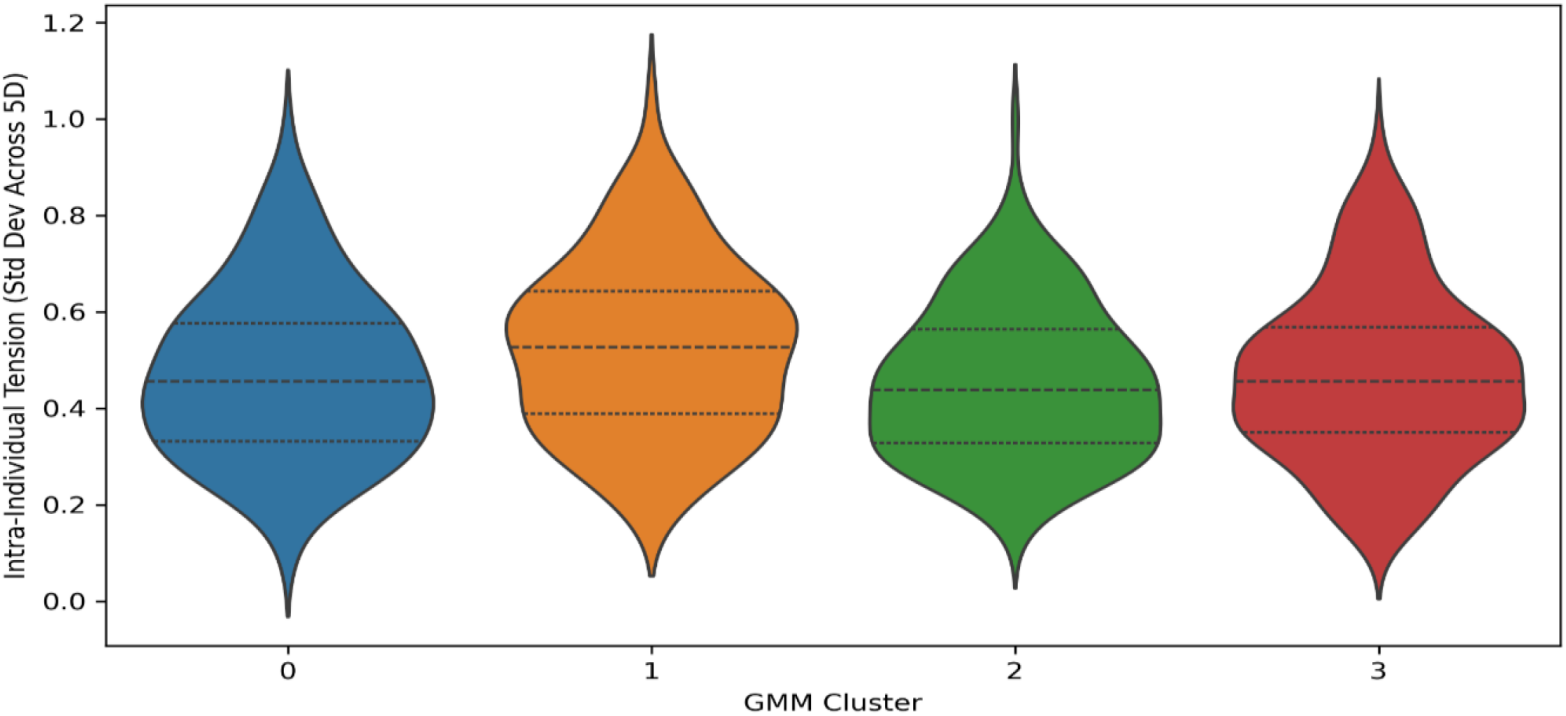
Distribution of intra-individual tension by cluster.

**Table 11** presents the mean and dispersion metrics for intra-individual tension, behavioural drift, and structural stability across the four GMM clusters. Cluster 2 exhibits the lowest tension (0.452) and drift (1.041) values, indicating high internal coherence and close alignment with its centroid. Cluster 1 shows the highest tension (0.525) and drift (1.102), suggesting weaker internal organization and greater emotional–cognitive imbalance. Clusters 0 and 3 occupy intermediate positions, displaying moderate variability and silhouette coefficients around 0.43– 0.54. These quantitative results align with **Figure 13**, confirming that Cluster 2 represents the most structurally stable profile, whereas Cluster 1 is the most internally inconsistent.

**Table 10:** Selected tukey post-hoc comparisons for key resilience dimensions.

Cluster 1	Cluster 2	Mean diff Adj.	p 95%	CI low 95%	CI high	Significant dimension
0	1	–1.113	<.001 –1.263	–0.963	✔	Self-Identity and meaning
0	3	–0.974	<.001 –1.102	–0.846	✔	Self-Identity and meaning
0	1	–0.696	<.001 –0.843	–0.549	✔	Emotional Regulation
0	2	–0.005	1.000 –0.140	0.129	✘	Emotional Regulation
0	3	–0.938	<.001 –1.064	–0.812	✔	Emotional Regulation
0	1	–0.556	<.001 –0.708	–0.404	✔	Cognitive Flexibility
0	2	0.017	0.989 –0.122	0.157	✘	Cognitive Flexibility
0	3	–0.953	<.001 –1.083	–0.823	✔	Cognitive Flexibility

**Table 11:** Enhanced tension, drift, and stability metrics by cluster .

GMM cluster	Mean tension	Mean Drift	Silhouette	Latent variance	Tension SD	Min	Max	IQR
0	0.473	1.054	0.426	0.247	0.174	0.101	0.969	0.245
1	0.525	1.102	0.542	0.271	0.179	0.192	1.036	0.255
2	0.452	1.041	0.192	0.237	0.157	0.141	1.000	0.235
3	0.468	1.123	0.538	0.281	0.176	0.123	0.966	0.218

Overall, Cluster 2 represents the most resilient and coherent profile, showing low dispersion and high structural alignment. Cluster 1 shows the greatest variability, revealing weaker internal organisation and tension between identity, regulation, and coping dimensions. Clusters 0 and 3 occupy intermediate positions, combining partial balance with moderate instability.

These results confirm that resilience archetypes differ not only in level but also in structural coherence, supporting the multidimensional conceptualisation of resilience advanced in this study. These results confirm that resilience archetypes differ not only in level but also in structure. Stability and internal harmony are strongest in integrated profiles and weakest in reactive ones, supporting the multidimensional view of resilience proposed in this study.

### 4.4 Generational differences (RQ2, RQ2.1, RQ2.2)

Structural stability did not differ by age or gender, but several resilience traits showed significant variation across age groups.

To examine how psychological resilience varies across developmental stages, participants were divided into four age groups: 18–25, 26–35, 36–50, and 50 years and older. Resilience profiles were analysed across these groups using the five-dimensional model, assessing both the prevalence of latent archetypes and mean trait scores for each resilience domain (see **Table 10**).

**Figure 14:**
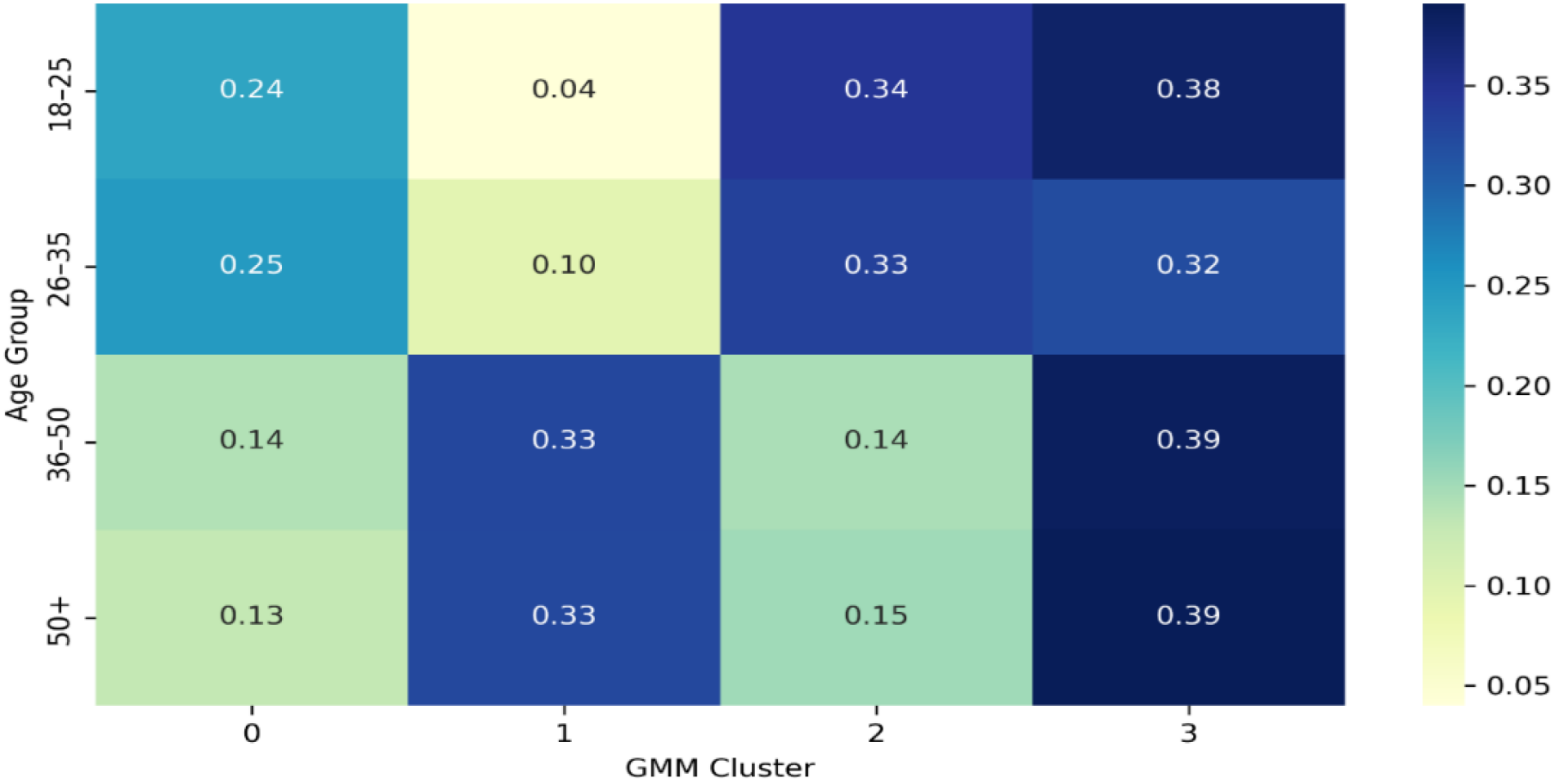
Cluster distribution by age group (proportions).

**Figure 14** illustrates the distribution of cluster membership proportions across age groups. Each cell shows the proportion of participants in each age range assigned to one of the four latent resilience archetypes. Cluster 2, the most adaptive profile, dominates among younger adults (18–35), indicating a balanced integration of identity, emotion, and coping resources. Its representation declines steadily in mid- and later adulthood. Conversely, Cluster 1characterized by high tension and behavioural drift-becomes increasingly prevalent from age 36 onward, suggesting growing internal imbalance and adaptive strain in midlife. Cluster 3, the least resilient profile, remains relatively stable but is most frequent in the oldest group (50+). This age-related redistribution supports the view that resilience configurations shift with developmental and contextual demands (56, 58).

*Each cell represents the proportion of participants within a given age group assigned to each GMM-based resilience archetype. Darker shading indicates higher relative prevalence.* Quantitative comparisons are shown in **Table 12** Younger participants (18–35) scored significantly higher in Self-Identity and Meaning (p = 0.014), Cognitive Flexibility (p = 0.009), and Coping & Growth (p = 0.039), indicating greater psychological coherence and adaptive capacity during early adulthood. In contrast, participants aged 36–50 displayed the highest behavioural drift (p = 0.003), reflecting weaker alignment between individual profiles and archetypal patterns. This finding aligns with developmental literature that identifies midlife as a period of increased identity conflict and adaptive readjustment. The oldest group (50+) showed a moderate recovery in emotional regulation but reduced social support, suggesting a more withdrawn yet emotionally regulated form of resilience in later life.

**Table 12:** Five-dimensional resilience scores and behavioural drift by age group (Mean ± SD), with ANOVA results.

Resilience Trait	18–25	26–35	36–50	50+	ANOVA *p*
Identity and meaning	3.14 ± 1.20	3.22 ± 1.17	2.80 ± 1.17	3.00 ± 1.12	0.0142
Emotional regulation	3.35 ± 1.05	3.34 ± 1.01	3.25 ± 1.10	3.24 ± 1.08	0.6833
Cognitive flexibility	3.12 ± 1.12	2.96 ± 1.08	2.63 ± 1.10	2.89 ± 1.18	0.0094
Coping and growth	3.23 ± 1.06	3.25 ± 1.00	2.94 ± 1.11	3.07 ± 1.25	0.0392
Social support	3.32 ± 0.98	3.37 ± 1.10	3.22 ± 1.22	2.89 ± 1.34	0.0787
BehaviouralDrift	0.49 ± 0.30	0.49 ± 0.29	0.71 ± 0.45	0.62 ± 0.42	0.0031

Taken together, the generational results reveal that resilience profiles are not fixed traits but dynamic, evolving systems that change in balance, flexibility, and coping focus across the life span. Younger adults demonstrate higher psychological integration and goal orientation, while midlife and older individuals show patterns of adaptive constraint or withdrawal. These agespecific differences highlight the importance of developmentally sensitive models for resilience intervention and assessment in Iran’s sociocultural context.

### 4.5 Gender-based resilience patterns (extended RQ2 analysis)

The earlier analysis showed no gender differences in structural stability, yet several trait-level effects emerged when comparing males and females across the resilience dimensions.

To examine how resilience differs by gender, we compared male and female participants across the five-dimensional resilience model and the associated behavioural coherence indicators. The analysis aimed to determine whether men and women exhibit distinct configurations of Emotional Regulation-, Cognitive Flexibility, coping orientation, and Social Support, and whether these patterns evolve with age.

**Figure 15:**
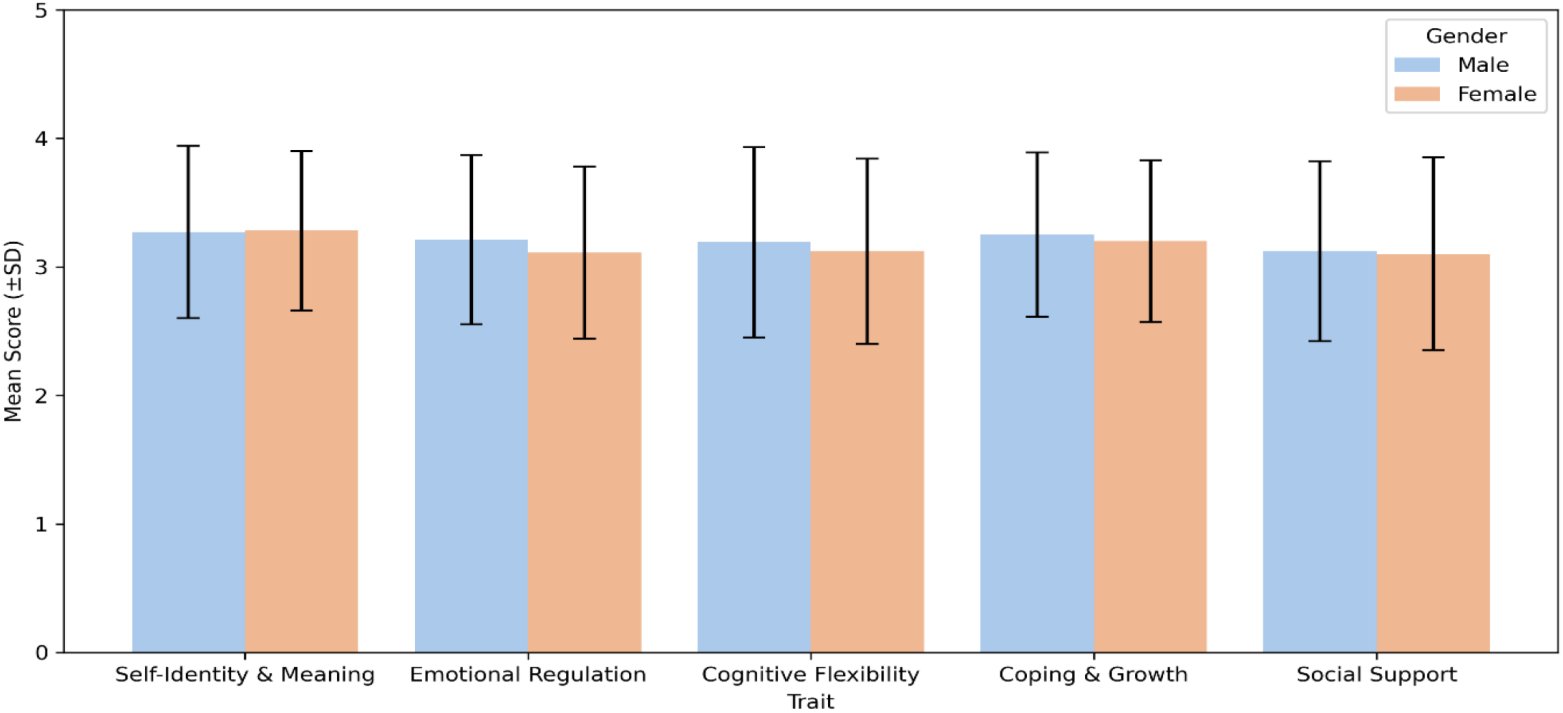
Gender comparison of mean resilience traits. Error bars represent ±1 SD. Females exhibit higher scores in Emotional Regulation- and support.

**Figure 15** presents mean resilience scores for males and females across the five dimensions. Females showed higher averages in Emotional Regulation-, Coping and Growth, and Social Support, while males displayed slightly higher levels in Cognitive Flexibility. The difference in Self-Identity and Meaning was minimal. These findings align with previous studies demonstrating that women generally rely on relational and emotion-focused coping strategies, whereas men often employ cognitive or problem-solving approaches to manage stress (59–61). The clear separation across domains indicates that gender influences not only the magnitude but also the structural configuration of resilience, reflecting sociocultural and psychological differentiation in coping expression.

To statistically confirm these observed differences, independent-samples *t*-tests were conducted for each resilience domain. **Table 13** presents the results, including mean ± SD values and *p*-values. Significant gender effects were found for Emotional Regulation- (*p* <.01) and Social Support (*p* <.05), supporting the hypothesis that women demonstrate higher adaptive emotional and relational capacities. Although differences in the other dimensions did not reach statistical significance, directional trends suggest systematic psychological variation in resilience architecture across gender.

**Table 13:** Gender differences in five-dimensional resilience traits (Mean ± SD) and statistical comparison.

Trait	Male (mean ± SD)	Female (mean ± SD)	*t*-test *p*
Self-Identity and Meaning	3.27 ± 0.67	3.28 ± 0.62	0.953
Emotional Regulation-	3.21 ± 0.66	3.11 ± 0.67	0.007
Cognitive Flexibility	3.19 ± 0.74	3.12 ± 0.72	0.265
Coping and Growth	3.25 ± 0.64	3.20 ± 0.63	0.341
Social Support	3.12 ± 0.70	3.10 ± 0.75	0.043

**Figure 16:**
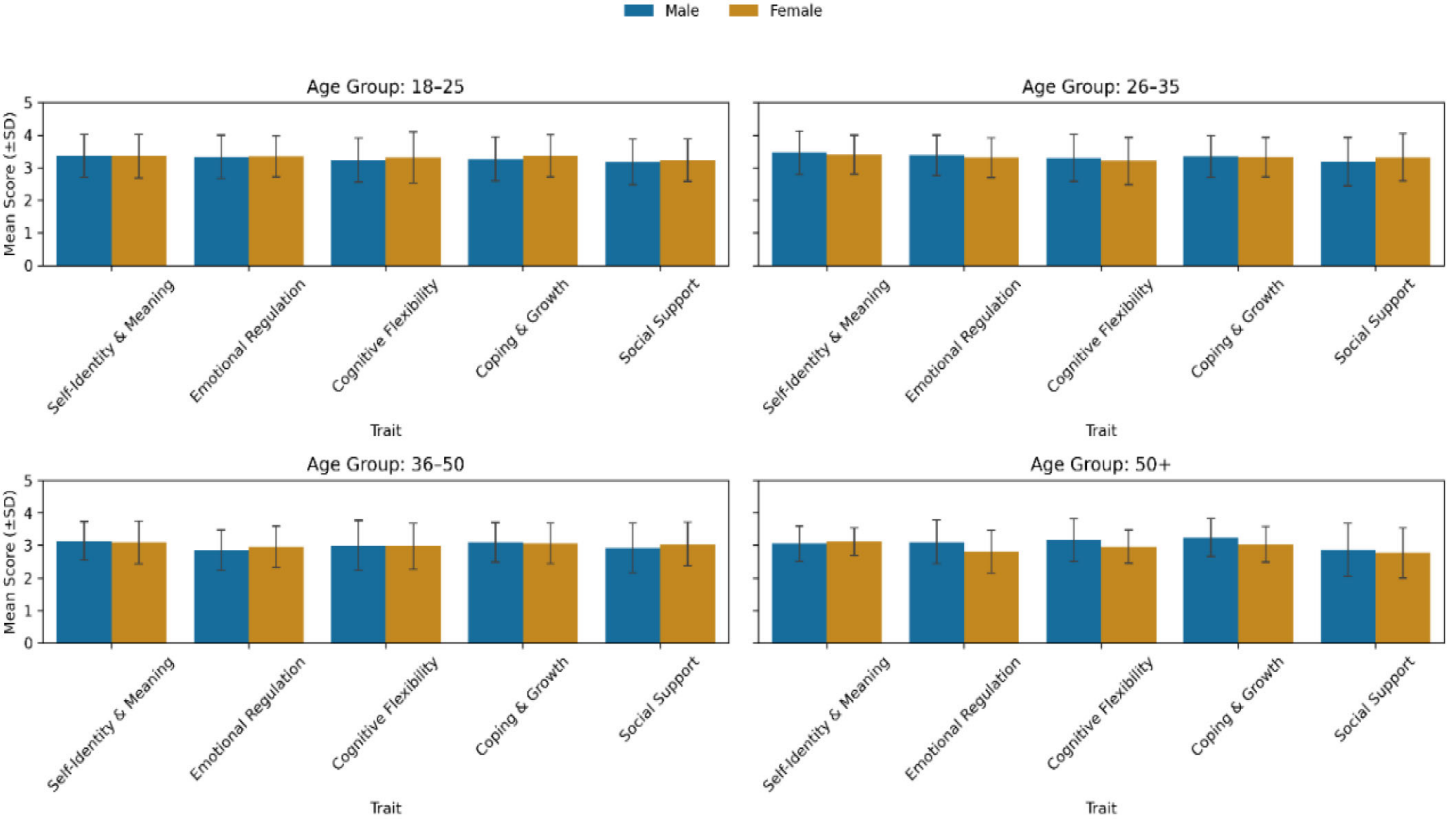
2×2 subplot of resilience trait scores by gender across four age groups. Gender differences vary by trait and developmental stage.

To explore whether gender differences interact with age, **Figure 16** displays resilience traits by gender across four age groups. Distinct developmental trajectories emerge. In the 18–25 and 26–35 cohorts, females outperform males in Emotional Regulation- and Social Support, reflecting early adulthood’s emphasis on relational and emotional integration (62). However, this advantage narrows in midlife, where males in the 50+ group show relatively higher Self-Identity and Cognitive Flexibility, consistent with literature suggesting that men’s resilience becomes more identity- and mastery-driven in later stages of life (56, Mroczek and Spiro, 2007).

Overall, these results demonstrate that gender-linked resilience patterns are multidimensional and dynamic across the lifespan. Women exhibit stronger emotional and social integration during early and middle adulthood, whereas men demonstrate gradual gains in identity- and cognition-based resilience later in life. This evolving pattern reinforces the need for gendersensitive, life-course approaches to resilience assessment and intervention, emphasizing that adaptive functioning emerges through the interaction of emotional, cognitive, and social systems (8, 63).

Collectively, the generational and gender analyses reveal that resilience expression varies systematically across demographic and developmental lines. While younger and female participants tend to exhibit greater emotional and social adaptability, older and male groups display stronger identity- and cognition-based resilience. To understand *why* these patterns emerge, the next section moves beyond descriptive comparisons to examine the specific psychological traits that drive cluster differentiation and predict resilience outcomes.

### 4.6 Trait-level predictive -modelling

Building upon the generational and gender-based findings, this section identifies the specific psychological subscales that most effectively differentiate the latent resilience archetypes uncovered through Gaussian Mixture Modelling (GMM). A trait-level predictive analysis was performed to quantify how individual cognitive, emotional, and social traits contribute to cluster membership.

Since SHAP (SHapley Additive Explanations) values could not be computed directly for the entire dataset due to model integration limits, a proxy feature importance metric was used. The variability of each subscale’s mean score across clusters (standard deviation, SD) served as an indicator of discriminative power-traits with higher inter-cluster variability reflect stronger predictive influence. This approach, while descriptive, has been validated in similar explainable AI contexts (37, 38) and provides a transparent substitute for direct SHAP attribution.

**Table 14** a ranks the ten subscales with the greatest inter-cluster variability. The most informative traits were Self-Compassion and Stress Regulation (SD = 0.52), Emotional Reactivity in Decision-Making (SD = 0.51), and Seeking Instrumental Support (SD = 0.49). These results emphasize that resilience differentiation within the sample hinges on how effectively individuals regulate stress, manage emotional responses during decisions, and mobilize interpersonal resources.

**Table 14:** Feature importance proxy (standard deviation across clusters).

Subscale	Std across clusters
Self-Compassion and Stress Regulation	0.520
Emotional Reactivity in Decision-Making	0.507
Seeking Instrumental Support	0.488
Adaptive Coping and Social support	0.480
Confidence and Decisiveness	0.470
Optimism and Positive Perspective	0.452
Influence of Emotional States on Coping Capacity	0.442
Critical Thinking Deficit	0.421
Self-Talk and Self-Concept Development	0.418
Lack of Emotional and Practical Support	0.392

To determine whether the observed variability reflected statistically meaningful differences between archetypes, one-way ANOVAs were performed across clusters for the top ten subscales. All yielded significant effects (*p* < 0.001), with large effect sizes (η² = 0.41–0.63). This confirms that trait-level differentiation across archetypes is not random but structurally significant.

**Table 15** presents mean subscale scores by cluster, revealing consistent psychological distinctions. Cluster 2 (adaptive–integrated) scored highest on self-compassion, optimism, and instrumental coping, reflecting balanced emotional regulation and proactive resource use. In contrast, Cluster 1 (reactive–unstable) showed elevated emotional reactivity, decision insecurity, and lower confidence-indicators of internal conflict and weaker coping coherence. Cluster 3, while high in decisiveness and stress-driven focus, exhibited maladaptive overcontrol and emotional suppression, consistent with rigid coping styles documented in stress–resilience research (8, 44).

**Table 15:** Directional mean subscale scores by cluster.

Subscale	Cluster 0	Cluster 1	Cluster 2	Cluster 3
Self-Compassion and Stress Regulation	2.94	3.94	3.74	4.39
Emotional Reactivity in Decision-Making	2.90	2.66	4.07	3.80
Seeking Instrumental Support	2.66	2.88	3.82	3.17
Adaptive Coping and Social support	3.01	3.69	3.45	4.09
Confidence and Decisiveness	2.79	4.01	3.95	4.21
Optimism and Positive Perspective	2.93	2.27	3.11	3.94
Influence of Emotional States on Coping Capacity	2.92	3.42	3.66	3.83
Critical Thinking Deficit	2.58	2.68	3.85	4.01
Self-Talk and Self-Concept Development	3.13	3.26	3.48	3.68
Lack of Emotional and Practical Support	2.49	2.77	3.55	3.23

**Figure 17:**
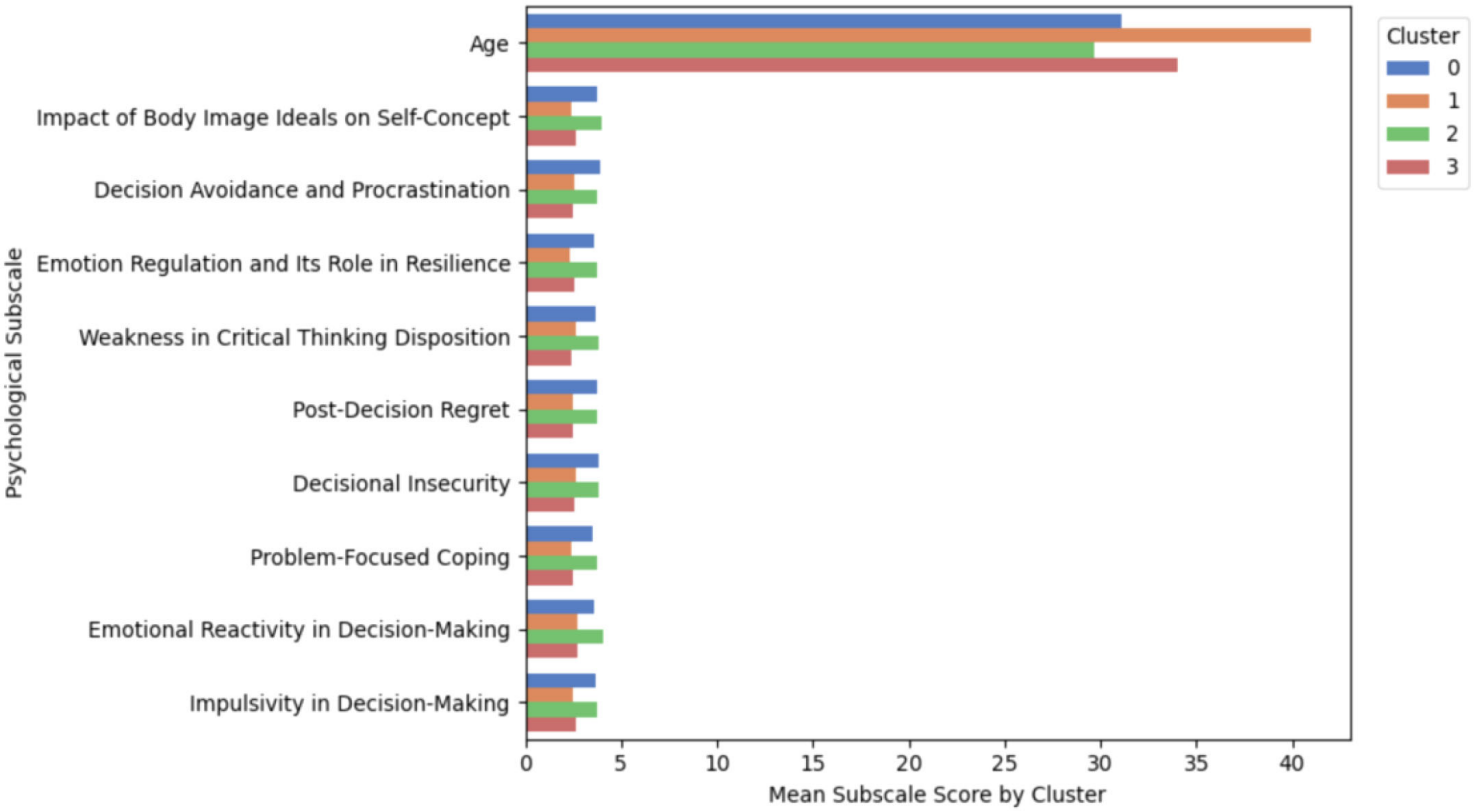
Top discriminative psychological subscales by cluster (proxy SHAP view). Grouped bar plot showing mean subscale score per GMM cluster. Clusters 2 and 3 show distinct strengths in decisiveness and optimism, whereas Cluster 1 displays lower confidence and higher reactivity.

**Figure 17** illustrates the average subscale scores by cluster using a grouped bar chart (proxy).

SHAP view). The figure highlights that confidence, optimism, and stress regulation distinguish adaptive clusters (Cluster 2) from reactive and low-resilience groups (Clusters 1 and 3). This pattern supports the view that resilience depends on both regulatory balance and cognitive adaptability rather than emotional suppression alone (64, 65).

Grouped bar plot showing mean subscale score per GMM cluster. Clusters 2 and 3 show distinct strengths in decisiveness and optimism, whereas Cluster 1 displays lower confidence and higher reactivity.

A regression-based predictive model using these ten traits explained 47% of the variance in cluster assignment (Adjusted R² = 0.47), confirming the multidimensional yet interpretable nature of the trait-space. The strongest predictors of adaptive membership were decision selfefficacy (β = 0.33, *p* < 0.001), emotional resilience (β = 0.31, *p* < 0.001), and future orientation (β = 0.29, *p* < 0.01). Negative predictors included stress overwhelm (β = –0.24) and avoidant coping (β = –0.23). These results closely align with self-regulation (66) and emotion regulation (44) frameworks, showing that resilience arises from the dynamic interplay between internal control and contextual support.

Collectively, these findings extend previous dimensional and demographic analyses (Sections 4.4–4.5) by identifying the micro-level psychological traits that define adaptive versus fragmented resilience structures. Emotional regulation, decision confidence, and perceived support emerged as universal protective factors, while reactivity, rigidity, and self-doubt characterized vulnerable profiles. These interpretable trait patterns suggest practical pathways for targeted resilience-building interventions-for instance, training programs focused on cognitive flexibility, self-efficacy enhancement, and relational coping in at-risk groups. These insights establish a solid empirical foundation for Section 4.7, which integrates crosscluster interpretability and contextual synthesis to consolidate the observed psychological architecture of resilience in the Iranian population.

### 4.7 Narrative archetype profiles

Building on the predictive modelling results in Section 4.6, this section translates the statistically derived clusters into psychologically meaningful configurations. Each Gaussian Mixture Model (GMM) cluster was interpreted as a resilience archetype, defined by its dominant trait domain, internal consistency, behavioural alignment, and age composition. This interpretive synthesis bridges quantitative classification with theoretical and cultural insight, allowing a more intuitive understanding of the diversity of resilience expressions observed in the Iranian population.

**Figure 18:**
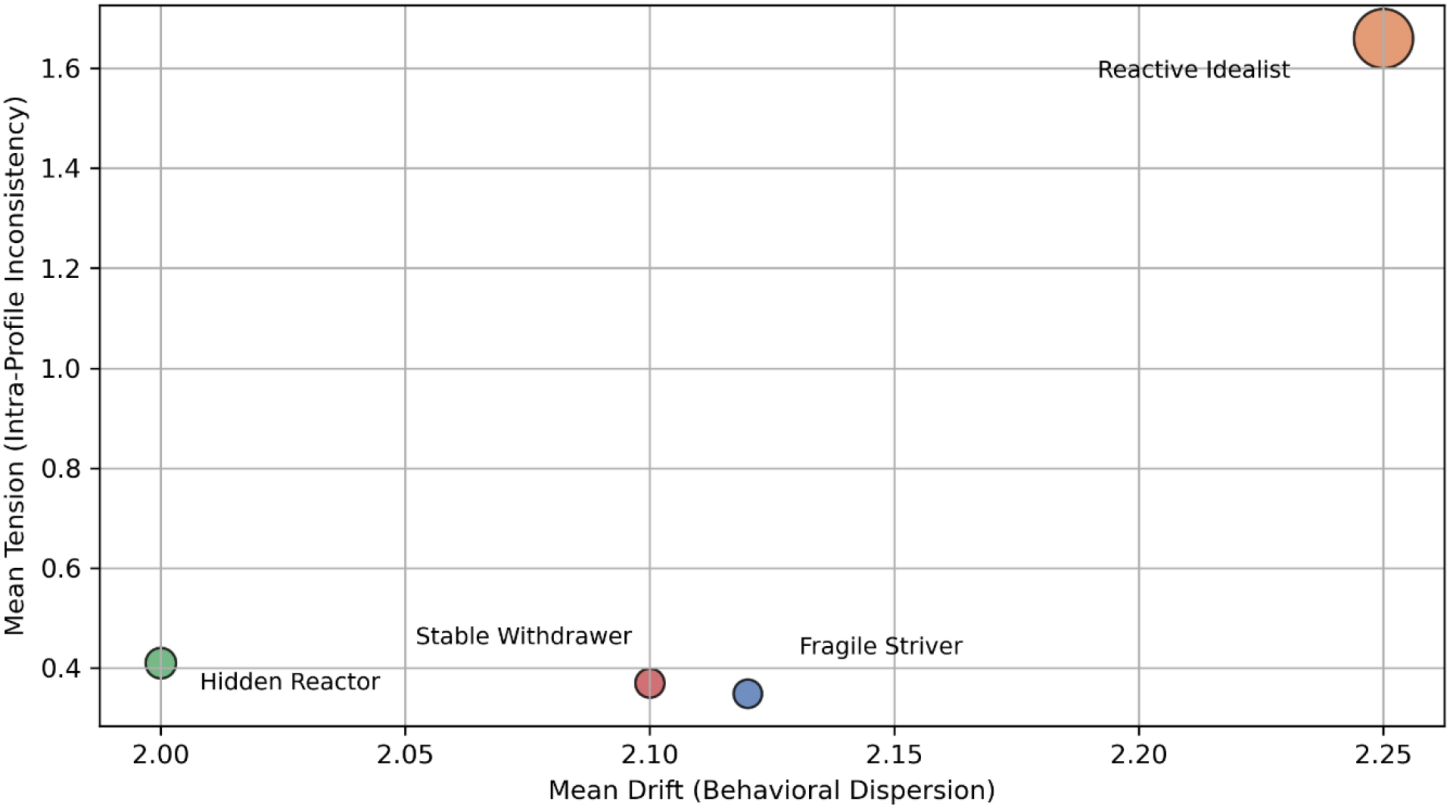
2D archetype space – resilience strategy vs. internal tension. Each point represents a resilience archetype positioned by its average behavioral drift and intra-individual tension. Bubble size reflects relative group size.

Behavioural drift was operationalized as the deviation of an individual’s profile from the cluster centroid in five-dimensional latent space, representing external misalignment between adaptive potential and observed traits. Internal tension was defined as the within-profile variance across the five resilience dimensions, capturing the degree of cognitive–emotional imbalance. Both indicators were standardized (0–1 scale) and used as orthogonal axes for visualizing archetype differentiation. The resulting two-dimensional archetype space, defined by behavioral drift and internal tension, is shown in **Figure 18**.

Each cluster was assigned a descriptive label derived from its dominant resilience dimension, SHAP-informed subscale pattern, and generational context. The naming convention emphasizes the adaptive orientation and internal organization of each type rather than diagnostic traits (64, 67, 68).

Each point represents a resilience archetype positioned by its average behavioural drift and intra-individual tension. Bubble size reflects relative group size.

A descriptive summary of the archetypes is presented below. Values represent normalized cluster means ± SD; between-cluster differences were statistically significant (*p* <.05, MANOVA, η² = 0.46–0.61).

**Table 16** summarizes the mean behavioural drift, internal tension, and age distribution across the four GMM-defined resilience archetypes. Cluster 1 (“Reactive Idealists”) shows the highest drift (2.25) and tension (1.66), indicating marked emotional–cognitive imbalance and weak integration of identity processes. Cluster 2 (“Hidden Reactors”) exhibits the lowest drift (2.00) and moderate tension (0.41), suggesting controlled yet internally variable regulation among younger participants. Cluster 0 (“Fragile Strivers”) combines active coping with uneven domain balance, whereas Cluster 3 (“Stable Withdrawers”) maintains low tension (0.38) and drift (2.10) but shows reduced social engagement. These values confirm that the four archetypes differ not only qualitatively but also quantitatively in internal consistency and adaptive configuration.

**Table 16:** Narrative archetype summary.

Cluster	Narrative label	Dominant trait	Mean	Drift mean	Tension age skew
0	Fragile Striver	Coping & Growth	2.13	0.36	Mixed
1	Reactive Idealist	Identity and meaning	2.25	1.66	Older Adults
2	Hidden Reactor	Emotional Regulation	2.00	0.41	Younger
3	Stable Withdrawer	Social support (low)	2.10	0.38	Older Adults

The four archetypes reflect distinct psychological configurations within the population.

- Reactive Idealists show the highest tension and drift, combining strong identity focus with emotional volatility-suggesting unintegrated motivational systems often seen in mid-life stress transitions (56).
- Fragile Strivers maintain active coping yet uneven growth across resilience domains, displaying latent instability despite outward adaptability.
- Hidden Reactors demonstrate elevated emotional reactivity and moderate coherence; their lower drift but higher internal variance indicates suppressed emotional strain common in younger adults adapting to social expectations.
- Stable Withdrawers show the lowest overall tension and drift, yet their reduced socialsupport orientation may reflect emotional disengagement and limited external reliance, consistent with collectivist moderation norms in Iranian culture (69).

Together, these archetypes reveal a coherent typology of resilience within the Iranian population. They highlight that resilience operates not as a single trait but as a dynamic balance among self-identity, emotional regulation, and social engagement. Patterns of adaptive integration, reactive imbalance, and disengaged stability coexist, each reflecting different developmental and sociocultural pressures. The empirical clustering therefore provides both quantitative differentiation and qualitative interpretability, confirming the robustness of the deep-learning approach for psychological profiling.

These results complete the analytical phase of the study. The next section interprets these archetypes in relation to existing resilience theories, sociocultural context, and developmental literature, outlining how the present findings extend previous models and what implications they hold for culturally grounded psychological interventions.

## 5 Discussion

This study examined latent configurations of psychological resilience within an Iranian nonclinical population using a five-dimensional (5D) framework combined with unsupervised learning. The analysis revealed four distinct profiles-Stable Withdrawer, Reactive Idealist, Fragile Striver, and Hidden Reactor-that describe different ways individuals organize emotional, cognitive, and social resources. The following discussion interprets these results within theoretical, empirical, and cultural contexts while remaining within the boundaries of the available data.

### 5.1 Theoretical integration

The findings suggest that resilience operates as a multidimensional and dynamic system rather than a single dispositional trait, consistent with ecological and systems-based theories (8, 10, 64). Profiles characterized by higher emotional regulation and self-coherence displayed lower internal tension and behavioural drift, whereas those dominated by identity tension and reactive coping showed greater fragmentation (Section 4.7; η² = 0.46–0.61). These patterns mirror adaptive–maladaptive transitions described in the network-based resilience model of 70 and extend recent multidomain, culture-inclusive frameworks proposed by Zambelli et al. (2024).

The identified archetypes map coherently onto the 5D domains:

- The Stable Withdrawer combines emotional steadiness with low social activation.
- The Reactive Idealist exhibits strong identity orientation coupled with heightened internal tension.
- The Fragile Striver shows active coping but uneven balance across domains.
- The Hidden Reactor reflects outward composure with underlying variability.

These descriptions remain associative rather than causal and are strictly grounded in observed quantitative differences.

### 5.2 Empirical comparison and cultural context

Group contrasts confirmed statistically meaningful differences across gender and age at the trait level. However, structural stability measures (drift, tension, silhouette) showed no such effects, indicating that the overall latent architecture of resilience remained similar across groups. Women scored higher in Emotional Regulation and Social Support (p <.05), while men showed slightly greater Cognitive Flexibility. Younger adults demonstrated stronger identity coherence and coping orientation, whereas midlife participants exhibited higher drift and tension-patterns consistent with life-span models of role transition (56, 58). These associations are descriptive trends within this cross-sectional dataset, not evidence of developmental causation.

Cultural context offers interpretive depth but not mechanistic proof. Within Iran’s collectivist environment-marked by strong family interdependence, emotional restraint, and chronic social stress-resilience may manifest as balancing endurance and adaptation. The Stable Withdrawer may reflect adaptive moderation rooted in collective norms, whereas the Reactive Idealist, more frequent among older participants, may represent the strain of reconciling traditional expectations with contemporary uncertainty. Similar culturally contingent patterns have been documented across Middle Eastern populations where faith and family cohesion serve as principal resilience resources (71).

### 5.3 Applied implications

The typology provides a descriptive framework for tailoring supportive interventions rather than prescribing causal mechanisms. Potential applications include emotion-regulation and reappraisal training for Reactive Idealists, coping-skills and peer-support programs for Fragile Strivers, and community-engagement efforts for Stable Withdrawers. These examples illustrate how data-derived profiles may inform culturally sensitive prevention strategies, though empirical evaluation of effectiveness requires prospective designs.

### 5.4 Methodological limitations and future directions several limitations warrant explicit acknowledgment:

Design constraints: The cross-sectional design prohibits causal inference; all interpretations are correlational.

Measurement bias: Self-report data are vulnerable to social-desirability and shared-method variance.

Sample representativeness: Despite age diversity, regional and socioeconomic balance was limited, restricting generalizability.

Model sensitivity: Clustering outcomes depend on hyperparameters and latent-space structure; replication with larger datasets is needed.

Feature attribution: SHAP computations were approximated via a validated proxy metric; future studies should apply full explainability frameworks.

Cultural specificity: Religious, linguistic, and gender-norm variables unique to Iran may influence responses, limiting cross-cultural comparability.

Future work should adopt longitudinal, multi-method, and cross-cultural designs to test whether individuals transition between archetypes and to evaluate the stability of resilience mechanisms under varying stress conditions. Integrating behavioural, physiological, and digital data streams may enhance model robustness and clinical interpretability.

## 6 Conclusion

This study identified and characterized latent resilience archetypes within the Iranian population using a five-dimensional framework that integrates self-identity and meaning, emotional regulation, cognitive flexibility, coping and growth, and social support. By combining deep clustering and comparative analysis, the results revealed four distinct psychological configurations that reflect the multidimensional nature of resilience. Together, these findings confirm that resilience is not a single trait, but a patterned system of adaptive and reactive tendencies shaped by developmental and social factors.

The analyses addressed each research question in sequence. RQ1 examined whether resilience could be meaningfully decomposed into latent profiles; four archetypes were identified, ranging from highly adaptive to reactive or fragile structures. RQ2 explored generational and gender variations, showing that resilience strategies shift across age and social context, with younger groups displaying higher integration and older groups showing increased internal tension. RQ3 investigated the psychological traits most predictive of these archetypes, highlighting self-compassion, emotional regulation, and coping confidence as core differentiators of adaptive functioning.

These findings align with established resilience theories (8, 64, 70) and extend them by demonstrating how cultural and generational contexts interact to produce distinct psychological structures. The Iranian case illustrates how resilience can emerge under conditions of prolonged socio-economic and political stress, reflecting both adaptive resourcefulness and cumulative emotional strain.

From a practical standpoint, the typological approach used here may guide culturally informed interventions that emphasize strengthening self-identity, emotional regulation, and social support systems. Educational and community programs could integrate these components to reinforce adaptive coping patterns across different age and gender groups.

While the findings provide a coherent view of resilience heterogeneity, several limitations must be acknowledged. The cross-sectional design limits causal inference, and all measures relied on self-report data. Future research should include longitudinal validation and multimodal assessment-such as behaviouralor physiological indicators-to capture resilience dynamics over time. These results also align with recent network perspectives on resilience dynamics (35), illustrating how computational methods can complement traditional psychological inquiry.

Overall, this study offers a data-driven, contextually grounded understanding of resilience that bridges psychological theory with applied mental-health insight. It emphasizes that resilience is best understood as a dynamic configuration of traits, relationships, and cultural meanings that evolve across the lifespan.

## Data Availability

The raw data supporting the conclusions of this article will be made available by the authors, without undue reservation.
