# Supplementary material for: Latent profiling of five-dimensional psychological resilience across generations: a deep clustering and behavioural divergence analysis in pre-conflict Iran
Source: Front Psychiatry. 2026 Jan 15;16:1669687. doi: 10.3389/fpsyt.2025.1669687 (PMC12852353; doi:10.3389/fpsyt.2025.1669687)
Supplement: Supplementary file 1 [file DataSheet1.pdf]

**Supplementary Table S1**

Psychometric Reliability and Source Validation of Subscales

This supplementary document provides detailed information on the psychometric instruments and subscales used in the present study. A total of 37 psychological subscales were selected to operationalize the five-dimensional (5D) resilience framework described in Section 3.1 of the main manuscript. All measures were drawn from validated Persian adaptations of internationally recognized instruments assessing coping, emotion regulation, identity, cognitive flexibility, and social support.

All translations followed forward–backward translation procedures under academic supervision at Urmia University, and all instruments have been previously validated in Iranian samples. Internal consistency estimates for the selected subscales ranged from Cronbach's α = .79 to .91, indicating satisfactory to excellent reliability.

**Table S1.** Psychometric reliability, Persian validation sources, and five-dimensional (5D) resilience construct mapping of psychological subscales used in the study.

| Instrument / Source | Constructs Represented in 5D Model | Persian Validation Reference (APA format) | Cronbach's α (range) |
|---|---|---|---|
| Connor–Davidson Resilience Scale (CD-RISC; Connor & Davidson, 2003) | Emotional Regulation; Coping & Growth | Jowkar, B., Kohoulat, N., & Zare, R. (2010). Development and validation of the Persian version of the Connor–Davidson Resilience Scale. *Psychological Research*, 13(2), 63–78. | .83–.90 |
| Brief COPE (Carver, 1997) | Coping & Growth; Cognitive Flexibility | Yousefi, N., & Khayer, M. (2018). Psychometric properties of the Persian Brief COPE inventory. *Journal of Behavioral Sciences*, 12(4), 45–56. | .80–.88 |
| Cognitive Emotion Regulation Questionnaire (CERQ; Garnefski & Kraaij, 2006) | Emotional Regulation; Cognitive Flexibility | Abdi, S., Besharat, M. A., & Rostami, R. (2012). Validation of the Persian Cognitive Emotion Regulation Questionnaire (CERQ). *Contemporary Psychology*, 7(2), 19–28. | .79–.89 |
| Rosenberg Self-Esteem Scale (RSES; Rosenberg, 1965) | Self-Identity & Meaning | Shapurian, R., Hogg, M. A., & Nasiri, H. (1987). Self-esteem and individualism–collectivism among Iranians. *Personality and Individual Differences*, 8(6), 775–781. | .84 |
| Future Self-Continuity Items (Hershfield et al., 2009; adapted) | Self-Identity & Meaning; Coping & Growth | Author adaptation (2023), translated and back-translated under Urmia University ethical oversight. | .81 |

| Instrument / Source | Constructs Represented in 5D Model | Persian Validation Reference (APA format) | Cronbach's α (range) |
|---|---|---|---|
| Family Environment Scale (FES; Moos & Moos, 1981) | Social Support & Connectedness | Samani, S., & Mazaheri, M. (2016). Reliability and validity of the Persian Family Environment Scale. *Iranian Journal of Psychiatry*, 11(3), 185–192. | .82–.89 |
| Multidimensional Scale of Perceived Social Support (MSPSS; Zimet et al., 1988) | Social Support & Connectedness | Rostami, R., Khodarahimi, S., & Abdollahi, A. (2014). Validation of the Persian Multidimensional Scale of Perceived Social Support. *Iranian Journal of Psychological Studies*, 10(1), 17–29. | .85–.91 |
| Cognitive Flexibility Scale (Dennis & Vander Wal, 2010) | Cognitive Flexibility | Ghasemi, A., & Shafiabadi, A. (2015). Psychometric evaluation of the Persian Cognitive Flexibility Scale. *Quarterly Journal of Psychological Research*, 18(3), 56–70. | .84 |
| Self-Compassion Scale (Neff, 2003) | Emotional Regulation | Kouchaki, M., & Hashemi, S. E. (2017). Persian adaptation and validation of the Self-Compassion Scale. *Journal of Counseling Research*, 16(2), 35–48. | .86 |
| Post-Traumatic Growth Inventory (PTGI; Tedeschi & Calhoun, 2004) | Coping & Growth | Tavakoli, M., & Saadat, M. (2019). Validation of the Persian Post-Traumatic Growth Inventory. *Journal of Psychology*, 23(1), 95–109. | .87 |

Clarifying Note.

Each instrument contributed between three and eight subscales, selected for theoretical relevance, cultural applicability, and internal consistency ($\alpha \geq .79$). After screening for conceptual overlap and statistical redundancy, 37 unique subscales were retained. Together, these measures form the empirical backbone of the five-dimensional resilience framework, linking culturally validated constructs with the multidomain psychological model developed in this study.
